# Tunable Osmium Complexes as pH-Insensitive Redox Reporters for Electrochemical Biosensing

**DOI:** 10.1021/acs.langmuir.6c02628

**Published:** 2026-08-12

**Authors:** Andrea Montón−Vicente, Jose Quilez−Alburquerque, Lorenzo Gramolini, Lucía Morillo−Victorero, Cristina García−Iriepa, Marco Marazzi, David Díaz Díaz, María Alba

**Affiliations:** † ARQUIMEA Research Center, San Cristóbal de La Laguna 38320, Spain; ‡ AFM-NANO, Instituto Universitario de Bio-Orgánica Antonio González (IUBO-AG), Universidad de La Laguna, Avda. Astrofísico Francisco Sánchez 2, La Laguna 38206, Spain; § Departamento de Química Orgánica, Universidad de La Laguna, Avda. Astrofísico Francisco Sánchez 3, La Laguna 38206, Spain; ∥ 98711 Universidad de Alcalá, Departamento de Química Analítica, Química Física e Ingeniería Química, Alcalá de Henares, Madrid 28805, Spain; ⊥ Institute of Chemical Research of Catalonia, Tarragona 43007, Spain; # Universidad de Alcalá, Instituto de Investigación Química “Andrés M. del Río” (IQAR), Alcalá de Henares, Madrid 28805, Spain

## Abstract

The lack of robust and tunable redox reporters continues to hinder the development of high-performance electrochemical biosensors. In this work, we present a combined computational and experimental investigation of a family of water-compatible Os­(II) complexes as pH-insensitive alternatives to the gold-standard methylene blue (MB). We systematically engineered, synthesized, and characterized, both computationally and electrochemically, four Os­(II) polypyridyl complexes. We achieved a broad formal potential (*E*_0_^′^) range from 0.0 to +0.34 V *vs* Ag/AgCl, while maintaining fast, quasi-reversible one-electron behavior. These trends were reproduced by theoretical calculations and linked to systematic changes in the highest occupied molecular orbital (HOMO) energy/metal–ligand charge distribution. Covalent immobilization of the complexes onto carboxylated self-assembled monolayers on gold via amide coupling underscored their suitability as redox reporters in electrochemical biosensors. The redox potential remained pH-invariant from acidic to basic conditions, in contrast to the pronounced shifts observed for MB-derived probes. Stability under rapid and continuous electrochemical pulses by square-wave voltammetry was also assessed, with complexes **1**, **2**, and **4** displaying high stability after 200 scans in buffer compared to complex **3** and MB-based probes. Collectively, these results provide design rules and establish Os­(II) complexes as modular redox reporters with on-demand features for advanced electrochemical sensors, including ratiometric and multiplexed sensing.

## Graphical abstract

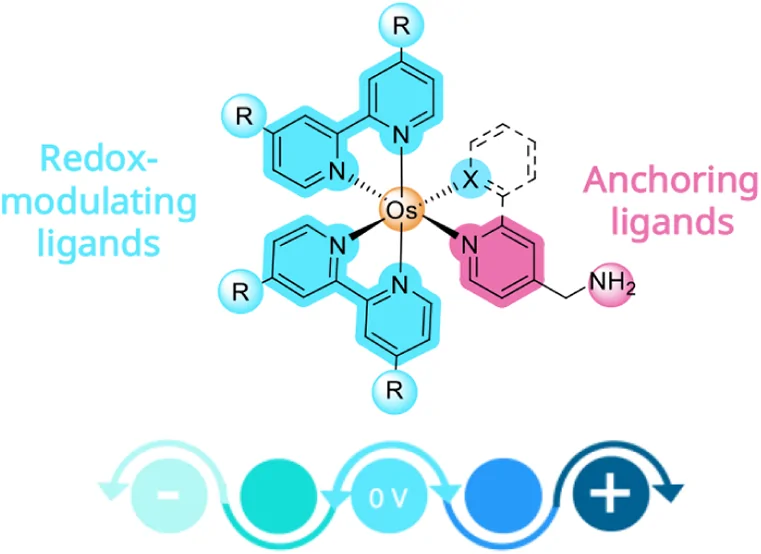

## 1 Introduction

Electrochemical biosensors have garnered significant attention over the past decades in a wide range of biotechnology-related sectors, including healthcare,^1^ food safety,^2^ and environmental monitoring.^3^ Their widespread relevance arises from their relative simplicity, which facilitates integration into electronic systems and miniaturization, together with their rapid response, high sensitivity, and selectivity.^4–6^ However, several challenges remain for the implementation of electrochemical biosensors in real-world applications, such as signal instability, matrix effects, and the scarcity of redox reporters that remain robust under continuous operation.^7^ In this regard, electrochemical biosensors’ performance can be noticeably enhanced by optimizing the electrode material and geometry,^8^ the immobilization strategy of the biorecognition element employed,^9,10^ the selection of an appropriate electrochemical technique,^11^ and the use of redox labels,^12^ among others. Although electrochemical biosensors have been extensively studied, progress is still limited by the narrow set of redox probes that fulfill the operational demands of field and clinical measurements.^13^

Among these platforms, electrochemical aptamer-based (EAB) sensors are especially sensitive to the choice of the redox reporter, as the redox label’s potential, electron-transfer kinetics, pH response, and cycling stability largely dictate signal gain and longevity. EAB sensors often employ thiol-modified aptamers chemically conjugated to the selected redox label and attached to a gold electrode via a self-assembled monolayer (SAM). Their operational principle relies on the conformational change of the aptamer upon recognition of its specific analyte, which alters the electron transfer between the tethered redox label and the electrode surface, ultimately leading to a variation in the electrochemical signal.^14^ Redox agents for EAB sensors must fulfill six critical features: (i) electrochemical reversibility; (ii) the capability to bind covalently to the oligonucleotide chain via cross-linking groups; (iii) a formal redox potential (*E*_0_^′^) within the suitable voltage window between −0.1 V to +0.4 V to avoid secondary redox processes such as oxygen reduction (*E* < −0.1 V) or gold oxidation (*E* > +0.4 V);^15^ (iv) pH insensitivity; (v) stability under continuous electrochemical cycling to enable real-time monitoring; and (vi) fast electron-transfer kinetics to maximize detectable changes in the electron transfer upon target binding.^16^

In practice, only a limited number of redox-active molecules are routinely used, including anthraquinone, Nile blue, methylene blue (MB), phenazine, and tetrathiafulvalene-based molecules, each fulfilling certain desirable properties but lacking in others.^17–20^ Among them, MB has been the most widely used, but its formal potential ( ${E_{0}}^{\prime }$ ≈−0.26 V vs Ag/AgCl) falls outside the desirable potential window, making it highly susceptible to oxygen reduction, elevating background currents, and undermining the stability of thiol-based monolayers on gold electrodes. Its redox reaction involves the exchange of one proton with the medium, making the probe pH-sensitive and limiting its versatility in matrices with varying pH, such as sweat and saliva.^16,21^ Alternatively, metal complexes—particularly Os­(II)—have attracted increasing interest in the field, offering compelling advantages over traditional organic redox labels. Indeed, osmium bipyridyl complexes and Os-containing redox polymers have a long history in bioelectrochemistry, where they have been widely used as mediators in enzymatic biosensors.^22,23^ Os­(II) complexes exhibit a *E*_0_^′^ that can be readily tuned into the aqueous “sweet spot” (≈−0.1 to +0.4 V *vs* Ag/AgCl), they are comparatively pH-insensitive, and they maintain signal stability under rapid pulsing. These attributes are valuable beyond EAB sensors, making them highly attractive for immuno-,^24^ multiplexed^25,26^ and ratiometric electrochemical sensors,^27,28^ where probes showing well-separated redox potentials are required. Thus far, few studies have been reported, essentially limited to the optimization of ferrocene by adding carbonyl groups to cyclopentadienyl ligands.^29^ Another promising alternative is an Os­(II) complex recently reported a potential redox probe for aptamers showing a pH-independent *E*_0_^′^ within the recommended voltage window and a high operational stability under continuous electrochemical pulses.^30^

Herein, we report the rational design and synthesis of a family of Os­(II) complexes as tunable redox reporters for electrochemical sensing interfaces, containing: (i) polyazaheteroaromatic ligands with different electronic substituents to finely tune their redox potentials; and (ii) an aminopyridine-based ligand for chemical conjugation. The complexes display well-resolved, reversible Os^2+^/Os^3+^ electrochemical characteristics with *E*_0_^′^ spanning from +0.036 to +0.340 V *vs* Ag/AgCl and small peak separations (Δ*E* = 0.08–0.14 V), positioning them within the aqueous “sweet spot” for sensing. Computational modeling reproduces and rationalizes these trends. For surface integration, the amine ligand supports carbodiimide-mediated amide coupling to carboxylate-terminated monolayers on gold. Surface immobilization allowed evaluation under conditions relevant to future biosensor architectures, the Os complexes retain their solution behavior and, critically, display pH-invariant redox potentials. Moreover, the complexes exhibit high stability under rapid, continuous square-wave interrogation.

## 2 Experimental Section

### 2.1 Chemicals and Materials

Ammonium hexachloroosmate (IV) ((NH_4_)_2_OsCl_6_), ammonium hexafluorophosphate (NH_4_PF_6_), sodium dithionite (Na_2_S_2_O_4_), 4-(aminomethyl)­pyridine (ampy), 4,4′-dimethyl-2,2′-bipyridine (dmb), 4,4′-dichloro-2,2′-bipyridine (dclb), 4,4′-dimethoxy-2,2′-bipyridine (dmob), (2-phenylpyridin-4-yl)­methanamine (ppyma), new methylene blue (NMB), 1-ethyl-3-(3-dimethylaminopropyl)­carbodiimide (EDC), guanidinium hydrochloride (GC), 11-mercaptoundecanoic acid (MUA), 6-mercapto-1-hexanol (MCH), hydrogen peroxide (30% H_2_O_2_ solution), sulfuric acid (H_2_SO_4_), sodium hydroxide (NaOH), sodium chloride (NaCl), magnesium chloride hexahydrate (MgCl_2_·6 H_2_O), phosphate buffer saline (0.01 mol L^–1^ PBS; 11.9 mmol L^–1^ Na_2_HPO_4_, 10.9 mmol L^–1^, KH_2_PO_4_, 1.9 mmol L^–1^, 137 mmol L^–1^ NaCl, 2.7 mmol L^–1^ KCl; pH 7.4), McIlvaine buffer (200 mmol L^–1^ Na_2_HPO_4_, 100 mmol L^–1^ citric acid) and Amberlite^TM^ IRA-900 Ion Exchange Resin (chloride form, 16–50 mesh), were purchased from Sigma-Aldrich and used without further purification. Ethylene glycol, acetonitrile, *N*,*N*-dimethylformamide (DMF), diethyl ether, dichloromethane (DCM), methanol (MeOH), and ethanol (EtOH) were purchased from VWR and used as received. Gold working electrode (Ø = 2 mm, CHI101), platinum wire counter electrode (CHI115), Ag/AgCl reference electrode (CHI111), 0.3 μm alpha alumina powder, 0.05 μm gamma alumina powder, nylon and microcloth polishing pads (CHI120 electrode polishing kit) were obtained from CH Instruments.

### 2.2 Instrumentation

Electrochemical characterization of the Os­(II) complexes was performed using a Multichannel potentiostat (OctoStat30) and hand-held potentiostat (pocketSTAT) from Ivium Technologies. UV–Vis absorption spectra were recorded with a HACH DR 6000 UV-Vis spectrophotometer. Mass spectra (ESI–MS) were measured with a Waters SYNAPT XS Q-TOF mass spectrometer with ion mobility.

### 2.3 Synthetic Procedure of Os­(II) Complexes

#### 2.3.1 Synthesis of [Os­(dmob)_2_Cl­(ampy)]­Cl (Complex **1**)

(NH_4_)­OsCl_6_ (200 mg, 0.456 mmol) and dmob (213 mg, 0.957 mmol) were reacted in 2.5 mL ethylene glycol at 100 °C under N_2_ atmosphere for 4 h. Reduction of Os^3+^ to Os^2+^ was accomplished with an aqueous saturated solution of Na_2_S_2_O_4_ (5 mL) in an ice bath to form a precipitate that was filtered and washed with water and diethyl ether to yield the intermediate complex *cis*-[Os­(dmob)_2_Cl_2_] as a dark solid (70% yield). This intermediate was used without further purification in the following step. *cis*-[Os­(dmob)_2_Cl_2_] (94 mg, 0.150 mmol) was reacted with ampy (18 mg, 0.16 mmol) in 6 mL of ethylene glycol, under N_2_ atmosphere at 120 °C for 4 h while stirring. The mixture was then allowed to cool down at room temperature for 30 min, and then 40 mL of a saturated aqueous NH_4_PF_6_ solution were added. The corresponding PF_6_ salt precipitated as a dark brown solid that was washed with cold water and diethyl ether and kept in a vacuum desiccator overnight. Alumina column was used for purification of the complex using DCM/MeOH as eluent (40% yield).

#### 2.3.2 Synthesis of [Os­(dmb)_2_Cl­(ampy)]­Cl (Complex **2**)

(NH_4_)­OsCl_6_ (300 mg, 0.683 mmol) and dmb (291 mg, 1.582 mmol) were reacted in 12 mL ethylene glycol at reflux under N_2_ atmosphere for 1 h. Reduction of the bis complex was accomplished by adding 10 mL of an aqueous saturated solution of Na_2_S_2_O_4_ in an ice bath to form a precipitate that was filtered and washed with water and diethyl ether to yield the intermediate complex *cis*-[Os­(dmb)_2_Cl_2_] as a dark solid (88% yield). This intermediate was used without further purification in the following step. *cis*-[Os­(dmb)_2_Cl_2_] (300 mg, 0.453 mmol) was reacted with ampy (60 mg, 0.557 mmol) in 9 mL of ethylene glycol, under N_2_ atmosphere at reflux for 2 h while stirring. The mixture was then allowed to cool down at room temperature for 30 min, and then 40 mL of a saturated aqueous NH_4_PF_6_ solution was added. The corresponding PF_6_ salt precipitated as a dark brown solid that was washed with cold water and diethyl ether and kept in a vacuum desiccator overnight (84% yield).

#### 2.3.3 Synthesis of [Os­(dclb)_2_Cl­(ampy)]­Cl (Complex **3**)

Complex **3** was synthesized by following literature methods and their NMR data were found to be consistent with those reported.^31^ Briefly, (NH_4_)­OsCl_6_ (150 mg, 0.342 mmol) and dclb (168 mg, 0.718 mmol) were reacted in 2.5 mL ethylene glycol at reflux under N_2_ atmosphere for 4 h. Reduction of Os^3+^ to Os^2+^was accomplished with an aqueous saturated solution of Na_2_S_2_O_4_ (10 mL) in an ice bath to form a precipitate that was filtered and washed with water and diethyl ether to yield the intermediate complex *cis*-[Os­(dclb)_2_Cl_2_] as a dark solid (62% yield). This intermediate was used without further purification in the following step. *cis*-[Os­(dclb)_2_Cl_2_] (94 mg, 0.15 mmol) was reacted with ampy (18 mg, 0.16 mmol) in 6 mL of ethylene glycol, under N_2_ atmosphere at 120 °C for 4 h while stirring. The mixture was then allowed to cool down at room temperature for 30 min, and then 40 mL of a saturated aqueous NH_4_PF_6_ solution was added. The corresponding PF_6_ salt precipitated as a dark brown solid that was washed with cold water and diethyl ether and kept in a vacuum desiccator overnight. Alumina column was used for purification of the complex using DCM/MeOH as eluent (42% yield).

#### 2.3.4 Synthesis of [Os­(dmb)_2_(ppyma)]­Cl (Complex **4**)

Complex **4** was synthesized by following literature methods and their NMR data were found to be consistent with those reported.^32^ Briefly, *cis*-[Os­(dmb)_2_Cl_2_] (82 mg, 0.15 mmol) was reacted with ppyma (35 mg, 0.16 mmol) in 3 mL of ethylene glycol, under N_2_ atmosphere at 120 °C for 5 h under stirring. The mixture was then allowed to cool down at room temperature for 30 min, and then 40 mL of a saturated aqueous NH_4_PF_6_ solution was added. The corresponding PF_6_ salt precipitated as a dark brown solid that was washed with cold water and diethyl ether and kept in a vacuum desiccator overnight. Alumina column was used for purification of the complex using DCM/MeOH as eluent (38% yield).

### 2.4 Electrode Cleaning and Electrochemical Measurement

2 mm Gold disk electrodes were first cleaned by submersion in piranha solution (1:3 H_2_O_2_–H_2_SO_4_) for 10 minutes. The electrodes were rinsed with deionized (DI) water before polishing for 2 min with 0.3 μm alumina slurry on a nylon polishing pad, followed by 5 min of polishing with 0.05 μm alumina slurry on a microcloth polishing pad. The freshly polished electrodes were electrochemically activated by scanning via cyclic voltammetry (CV), first from −0.5 to −1.0 V in 0.1 mol L^–1^ NaOH (20 cycles), then from −0.1 to 1.6 V in 0.5 mol L^–1^ H_2_SO_4_ (at least 40 cycles), at a scan rate of 0.1 V s^–1^ and voltage step of 5 mV. After this process, the electrodes were ready for functionalization. For electrochemical measurements, a glass electrochemical cell was assembled with a gold disk working electrode, an Ag/AgCl (3 mol L^–1^ NaCl) reference electrode, and a Pt counter electrode. Unless otherwise stated, all square wave voltammograms of Os­(II) complexes in this work were measured using a square wave amplitude of 25 mV, a voltage step of 1 mV, and a frequency of 500 Hz. Cyclic voltammograms of Os­(II) complexes were measured using a scan rate of 5 V s^–1^ and a voltage step of 1 mV. NMB square wave voltammograms were measured using an amplitude of 25 mV, a voltage step of 8 mV, and a frequency of 25 Hz. NMB cyclic voltammograms were recorded at 250 mV s^–1^ with a 5 mV potential step. Electrochemical data from CV and square wave voltammetry (SWV) experiments were acquired and processed using IviumSoft software (Ivium Technologies), approximated by the experimental half-wave potential (E_1/2_), was determined as the average of the anodic and cathodic peak potentials. All measurements were performed at least in triplicate under identical conditions. Statistical analyses and graphical representations were performed using GraphPad Prism 10 and the Standard Error of the Mean (SEM) to measure the samples’ variability.

### 2.5 Preparation of Functionalized Gold Electrodes and Attachment of an Os­(II) Complex

The functionalization procedure was adapted from a previously reported method,^30^ with slight modifications. A pretreated gold electrode surface was modified with a MUA monolayer by incubation in an ethanolic solution containing 1 μmol L^–1^ of MUA for 2 h. The electrode was washed sequentially with EtOH and DI water. The Os­(II) complex was covalently attached to the carboxylic-terminated MUA monolayer by immersing the electrode overnight in an aqueous solution containing 30 mmol L^–1^ EDC, 5 mmol L^–1^ MgCl_2_·6 H_2_O, 150 mmol L^–1^ NaCl, and 0.1 mmol L^–1^ of the corresponding Os­(II) complex. To remove any nonspecifically adsorbed Os­(II) complex from the gold surface, the electrode was subjected to two consecutive washing steps: first, immersion in a 1 mol L^–1^ H_2_SO_4_ solution in acetonitrile for 30 min; then, the electrode was rinsed with DI water, and it was introduced in a 6 mol L^–1^ GC solution in 4:1 v/v EtOH/DI water for another 30 min. Finally, after rinsing with DI water, the electrode was incubated overnight in 10 mmol L^–1^ MCH for backfilling of the monolayer.

### 2.6 Computational Methods

The level of theory employed in the Time Dependent Density Functional Theory (TD-DFT)^33,34^ calculations were determined through benchmarking of functionals and basis sets previously reported in the literature for analogous metal complexes.^35–42^ To avoid biasing the benchmarking process toward any of the specific complexes under investigation, [Os­(bpy)_3_]^2+^ (where bpy is 2,2-bipyridine) was selected as a reference, as it is chemically similar to all four Os­(II) complexes under examination, and its extensively characterized absorption spectrum is in the literature.^43–45^ The same computational method was subsequently applied to all the complexes studied. Ground-state geometries of the *cis* isomers were optimized by DFT in the presence of water using the Integral Equation Formalism Polarizable Continuum Model (IEFPCM)^34^ as an implicit solvation model, and subsequent frequency calculations were performed. Finally, the effect of substituting the chloride ligand with other species was evaluated. All calculations were performed with the Gaussian16 software.^46^

## 3 Results and Discussion

### 3.1 Rational Design of Os­(II) Complexes as Potential Redox Probes

In this work, we rationally designed and synthesized four different Os­(II) complexes as candidate redox probes for electrochemical sensors. The systematic design of the Os­(II) complex structures was based on the judicious selection of surrounding ligands coordinated to the metal center to modulate their electronic properties. For this purpose, electron-donating moieties such as methoxy or methyl groups (complexes **1** and **2** in Figure 1, respectively) and electron-withdrawing moieties such as chlorine (complex **3** in Figure 1), were introduced on bipyridyl-based ligands to assess the inductive electronic effect of these substituents on the resulting *E*_0_^′^ value. To explore whether replacing the free chlorine ligand with a carbon-metal bond further decreases the redox potential of these polypyridyl Os­(II) complexes^47^ in comparison with their homoleptic analogs, we prepared Os­(II) complex **4** (Figure 1) featuring a carbometalated phenylpyridine ligand. To enable attachment to SAMs on electrode surfaces, all complexes contain a free amine group tethered to a pyridine (complexes **1**, **2**, and **3**) or phenylpyridine (for complex **4**) ligand, facilitating coupling to carboxylic acid functionalized surfaces via amide bond formation.

**Figure 1:**
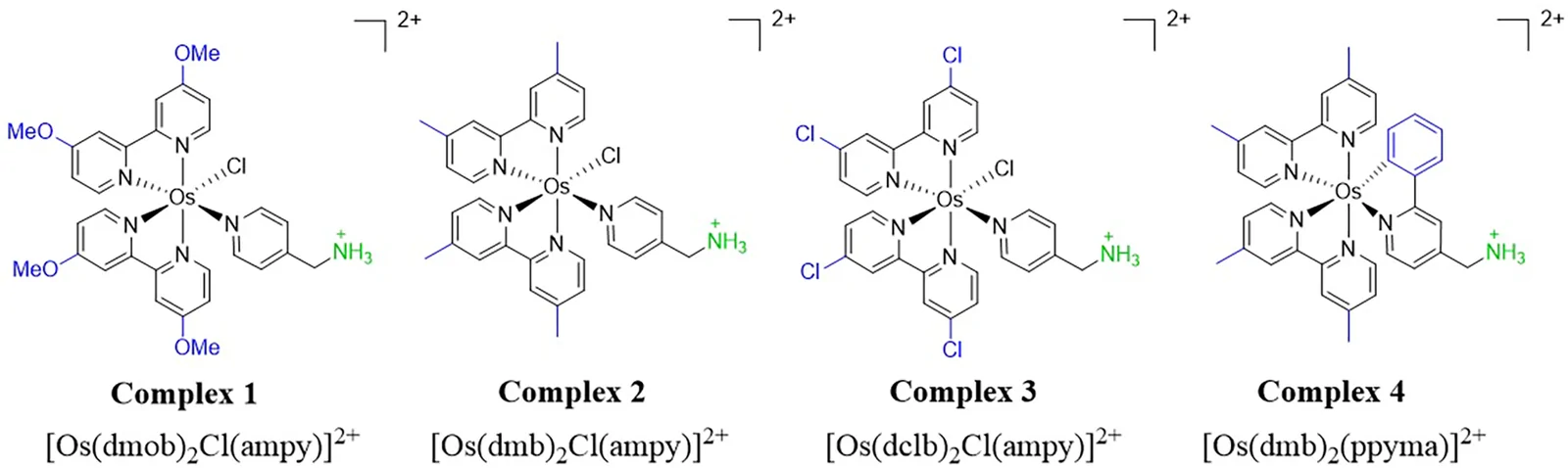
Chemical structures of the Os­(II) complexes 1–4 designed as tunable redox probes for electrochemical sensing.

The redox probes were synthesized via a two-step route following the Scheme S1 (Supporting Information, SI), based on previously established protocols with modifications.^48,49^ Briefly, the *cis*-Os­(NN)_2_Cl_2_ precursors were obtained by reacting the chelating ligand with (NH_4_)_2_OsCl_6_, followed by reduction with Na_2_S_2_O_4_ to obtain the intermediate Os­(II) bis complexes. The target Os­(II) probes were synthesized by reacting the corresponding *cis*-Os­(NN)_2_Cl_2_ and ampy or ppyma. Figures S1-S9 in the SI show the structural confirmation of the synthesized Os­(II) redox probes by ESI-MS and ^1^H-NMR. Finally, all the Os­(II) complexes were converted to chloride salts using a chromatographic column charged with Amberlite^TM^ IRA-900 ion exchange resin to enhance water solubility. While complex **4** has a unique structural configuration, complexes **1**, **2**, and **3** can exist as *cis* and *trans* isomers. However, calculations showed that the *cis* isomer is more stable in all cases by more than 10 kcal mol^–1^ (Figure S10, SI). Consequently, at room temperature, the *cis* isomer is expected to be the predominant structure for all the complexes (Figure 1). The difference in Gibbs free energy at 25 °C relative to the *trans* isomer was also calculated (Figure S10, SI), supporting the negligible contribution of the *trans* form.

### 3.2 Spectroscopic Characterization of the Os­(II) Complexes

The experimental and calculated UV-Vis absorption spectra of the Os­(II) complexes in aqueous solvent are depicted in Figure 2 and summarized in Table 1. The spectra were calculated using TD-DFT,^50^ with [Os­(bpy)_3_]^2+^ employed as the reference. The benchmarking results (Figures S11 and S12, SI) supported the selection of the B3LYP functional,^51^ with a def2-TZVP basis set for the osmium atom (including the pseudopotential for core electrons), and the def2-SVP basis set for the remaining atoms.^52,53^

**Figure 2:**
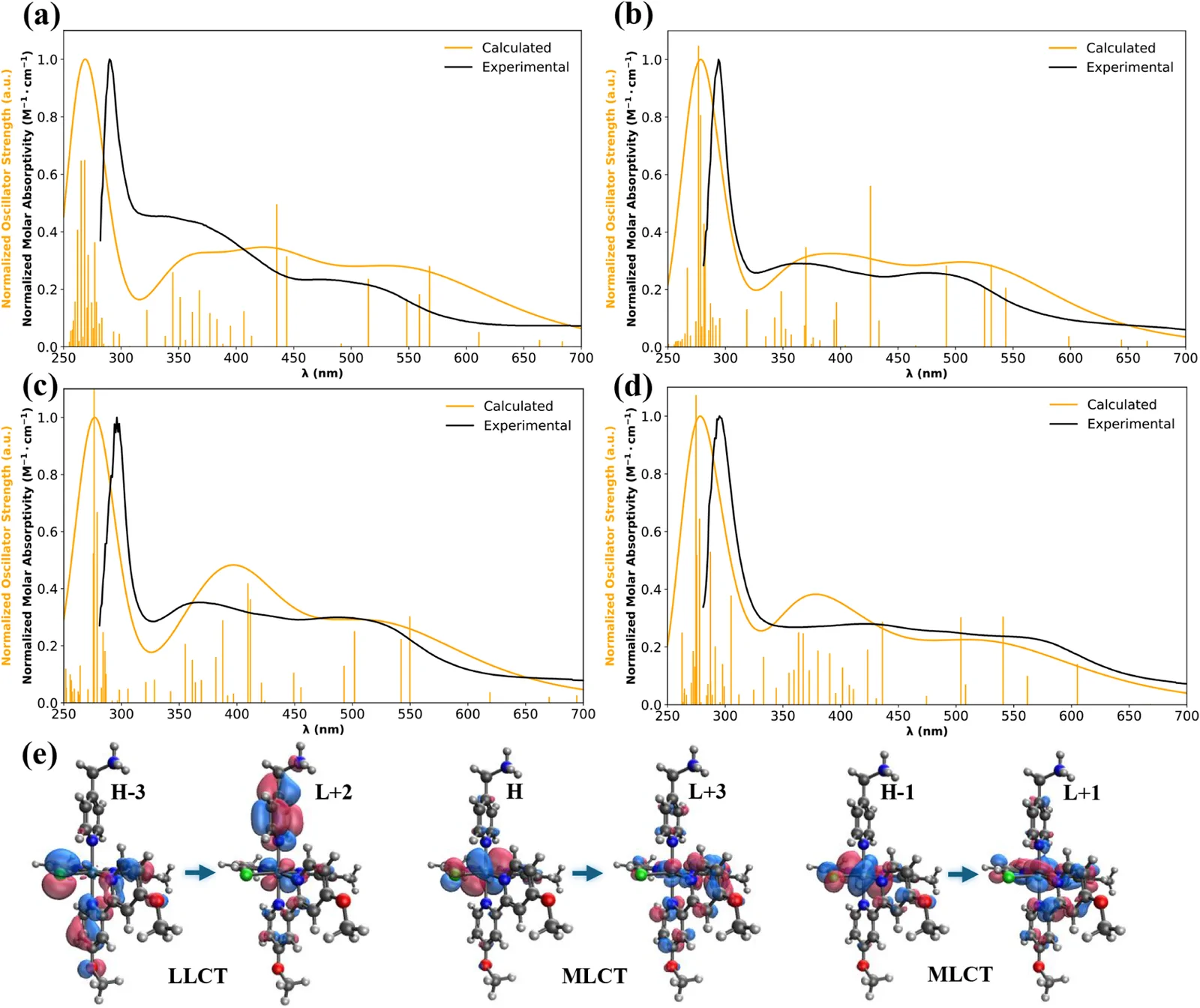
Calculated (orange) *vs* experimental (black) UV-Vis absorption spectra in water and PBS, respectively, of (a) complex **1**, (b) complex **2**, (c) complex **3**, and (d) complex **4**. Orange vertical sticks indicate the individual computed transitions; orange curves are the corresponding convoluted envelopes. All spectra are normalized to their most intense band. (e) Orbitals involved in the main electronic transitions for complex **1**, with H and L denoting HOMO and LUMO, respectively. LLCT corresponds to the most energetic peak; MLCT corresponds to the middle and less energetic peaks of the absorption spectrum.

**Table 1:** Experimental and Calculated (*calc*) Absorption and Redox Potential Values^a^

Os(II) complex	λ_abs_^max^/nm (ε/10^3^ mol L^–1^ cm^–1^)	λ_abs_^max,calc^/nm (ε/10^3^ mol L^–1^ cm^–1^)	*E*_1/2_ /V	*E*_1/2_^calc^ /V	Δ*E* ^b^ /V
**1**	290 (4.4), 350 (2.4), 500 (1.2)	269 (4.4), 371 (1.5), 534 (1.2)	0.036	–0.031	0.12
**2**	294 (23.4), 373 (9.4), 483 (8.8)	279 (23.4), 390 (7.7), 513 (7.0)	0.195	0.129	0.08
**3**	297 (13.6), 366 (8.4), 512 (6.9)	277(13.6), 397 (6.5), 512 (3.9)	0.340	0.339	0.14
**4**	294 (28.1), 422 (7.9), 565 (6.5)	278 (28.1), 378 (11.0), 518 (6.5)	0.089	–0.12	0.08

a Experiments were performed in air equilibrated PBS (pH 7.4) solutions at 25 ± 0.1 °C. Calculations were performed in water as an implicit solvent at 25 °C.

b Δ*E* = *E*_ox_^CV^ – *E*_red_^CV^.

Overall, the calculated spectra qualitatively reproduced the spin-allowed singlet-singlet transitions experimentally observed in the UV-Vis profiles for complexes **1–4** (Figure 2a-d; Table 1). In all cases, three main peaks were observed: an intense band slightly below 300 nm assigned to ligand-to-ligand charge-transfer (LLCT) states, and two broader, less intense bands in the 350–570 nm region—extending into the visible range—both of metal-to-ligand charge-transfer (MLCT) character. As expected, the strong spin-orbit coupling associated with the heavy atom (Os) effect tends to broaden the MLCT band and gives rise to spin-forbidden singlet-triplet transitions that commonly fall in the Vis to near-IR region.^54,55^ Furthermore, the loss of symmetry in the complex further diminished the well-structured band maxima.^56^ Nevertheless, because spin-orbit coupling was not explicitly included in the calculations, only the most intense spin-allowed singlet-singlet transitions can be directly compared with the experimental data. Substitution of one bpy ligand in the reference [Os­(bpy)_3_]^2+^ caused a bathochromic shift of the MLCT. For complexes **1–3**, the MLCT bands shifted from 479 nm for [Os­(bpy)_3_]^2+^ (Figures S11 and S12, SI) to 483 nm for complex **2** (−Me), 500 nm for **1** (−OMe) and 512 nm for **3** (−Cl) (Figure 2a-c; Table 1). It should be noted that a slight discrepancy between theory and experiment is observed regarding the intensity of the highest energy MLCT band of complex **1**. This difference is mainly attributable to the broadening of the calculated MLCT band and to the concomitant increase in the experimental intensity induced by electron-donating methoxy groups in the near UV-region, as observed for this type of complex.^57^ In complex **4**, the substitution of one bpy ligand by the carbometalated ppyma caused a drastic bathochromic shift, with an MLCT maximum at 565 nm (Figure 2d; Table 1), consistent with stronger σ-donation upon C-Os bond formation.^58^ A similar trend was also observed by modulating the electronic features of the ancillary ligands (Table 1). Electron-donating substituents (−Me; −OMe) decreased the *d* → π*­(ligand) back-donation (due to the higher energy of their π* orbitals), increasing the energy of the metal *t*_2g_^π^ orbitals, whereas electron-withdrawing substituents (−Cl) increased the *d* → π* (lowering π*) and stabilized the metal *t*_2g_^π^ orbitals.^59,60^ Both effects shrank the electronic transition energy, causing a red shift of the MLCT band compared to [Os­(bpy)_3_]^2+^. The orbital plots in Figure 2e illustrate these assignments for complex **1** (analogous diagrams for complexes **2**–**4** are provided in Figure S13, SI).

### 3.3 Electrochemical Characterization of the Os­(II) Complexes

To study the effect of the coordinating ligands on the *E*_1/2_ value of the complexes, we recorded cyclic voltammograms in PBS of complexes **1**–**4** (Figure 3). In each case, the first scan was initiated from the open-circuit potential (OCP) to assess the initial redox state and to screen for electroactive impurities, and is shown in gray. We have defined an optimal window for each complex to avoid secondary redox process as gold oxidation or oxygen reduction (see Figure S14 in the SI). The first OCP-initiated scan showed the expected Os­(II/III) redox couple without additional redox peaks or shoulders, supporting the absence of significant electroactive impurities detectable by CV. The 10th scan, shown as a representative stabilized response after repeated cycling, retained the same redox couple, indicating stable voltammetric behavior. As shown in Figure 3, the Os­(II/III) redox couple shifted systematically to more positive potentials from complexes **1** to **3**, consistent with the increasing electron-withdrawing character of the substituents. The symmetry of the peak areas, as well as the low peak-to-peak separation (Δ*E* = 0.08–0.14 V; Table 1) observed for each Os­(II) complex, indicated quasi-reversible one-electron behavior and rapid electron transfer between the electrode and the complexes. The concentrations used for the solution-phase CV measurements were adjusted for each complex to obtain well-defined and comparable voltammetric responses (1.25, 0.052, 0.23, and 0.18 mmol L^–1^ for complexes 1–4, respectively). Therefore, the absolute peak currents in Figure 3 should not be interpreted quantitatively across the series. Nevertheless, these factors do not alter the overall voltammetric profile, as all complexes exhibit the characteristic shape and redox features of the Os^2 +^ /Os^3 +^ couple. The recorded *E*_1/2_ values (*vs* Ag/AgCl) fall within a compact, sensing-friendly window and followed the expected substituent trend: complex **1** (−OMe, +0.036 V) < complex **2** (−Me, +0.195 V) < complex **3** (−Cl, +0.340 V), while the carbometalated complex **4** exhibited an *E*_1/2_ of 0.089 V, comparable to complex **1** despite their structural differences (Table 1).

**Figure 3:**
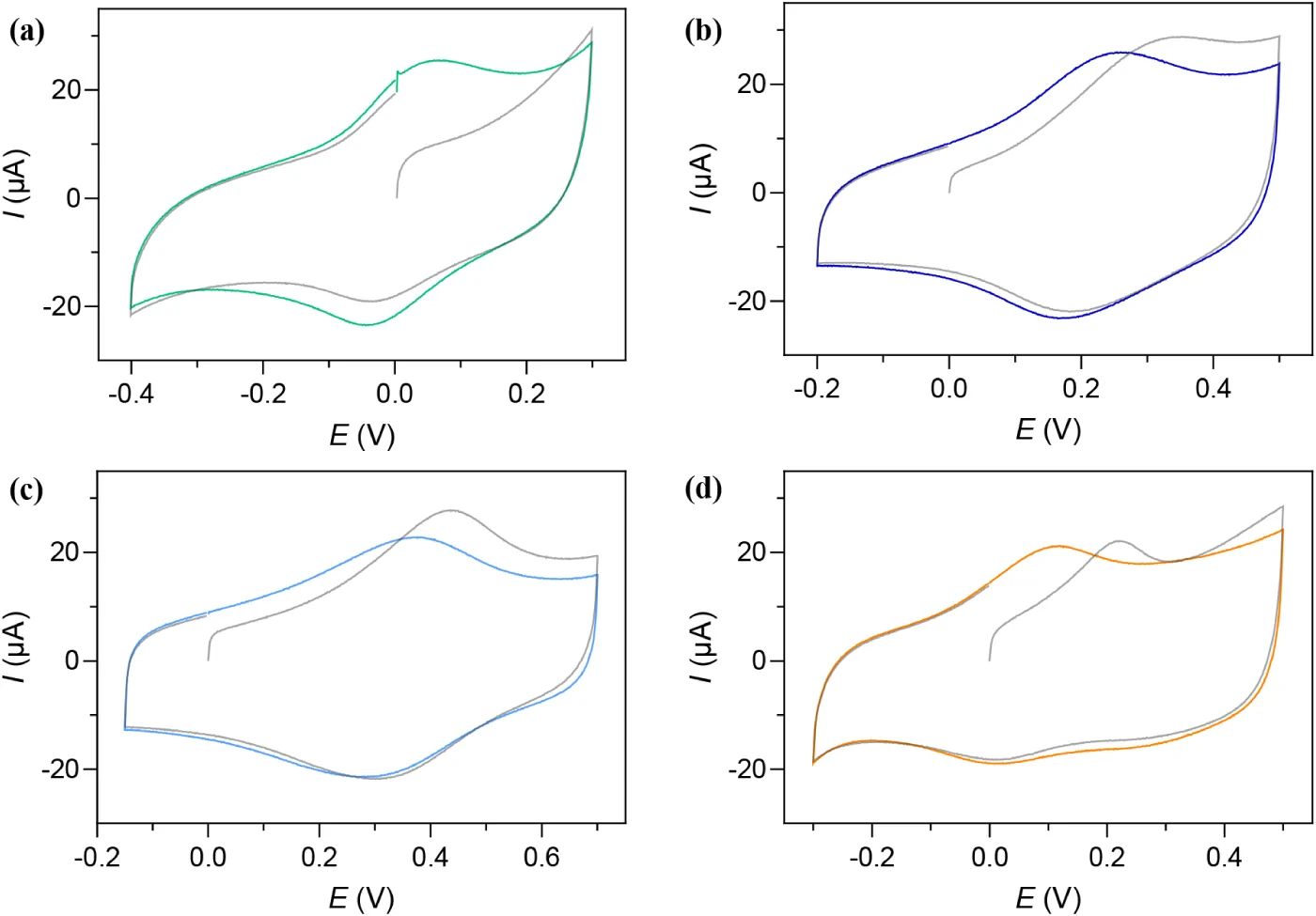
Cyclic voltammograms of complexes **1**–**4** in PBS (pH 7.4) of (a) complex **1** (1.25 mmol L^–1^), (b) complex **2** (0.052 mmol L^–1^), (c) complex **3** (0.23 mmol L^–1^), and (d) complex **4** (0.18 mmol L^–1^). The first scan, initiated from the OCP, is shown in gray, while the 10th scan is shown in color. All measurements were collected using a 2 mm diameter gold disk working electrode, at a scan rate of 5 V s^–1^ and a potential step of 1 mV.

To gain insights into the influence of the ancillary ligands, we computed their *E*_1/2_ values. The absolute redox potentials were calculated according to the formula *E* ^*0*^_abs_^calc^ = Δ*G*/*nF*, where Δ*G* is the difference in Gibbs free energy between oxidized and reduced species, *n* is the number of electrons involved in the oxidation/reduction process (1 electron in this study), and *F* is the Faraday constant. Accordingly, optimized geometries and vibrational frequencies were computed for the oxidized forms of the complexes to obtain their Δ*G* value. For the sake of comparison, the calculated redox potentials were expressed against the Ag/AgCl reference electrode using *E*_1/2_^calc^ = *E* ^0^_abs_^calc^ – 4.637 V (Table S1, SI).^61,62^ First, theoretical calculations were conducted to evaluate the effect of replacing the equatorial chloride ligand with other species present in the measuring solution (H_2_O and H_2_PO_4_^–^ from PBS buffer), as the chloride metal bond could be particularly labile in aqueous media.^63^ The resulting *E*_1/2_^calc^ values for the different alternatives confirmed that the chloride was the most likely ligand bound to the complex (Table S1, SI), thereby supporting the assigned structure of Os­(II) complexes **1**–**3**.

The redox potential of complexes **1**–**4** is intrinsically correlated to the energy of the metal *t*_2g_^π^ orbitals (HOMO energy). While σ-donor chelating ligands increase the energy of these orbitals, favoring the oxidation (↓*E*_1/2_), π-acceptor ligands stabilize these orbitals (↑*E*_1/2_). Figure 4 and Table 1 summarize these trends for complexes **1**–**4** and [Os­(bpy)_3_]^2+^, used as a reference. The substitution of one bpy ligand in [Os­(bpy)_3_]^2+^ by one ampy or ppyma clearly increased the HOMO energy, confirming the redox modulation within the optimal window for biosensing applications. In detail, the coordination of carbometalated ligands (ppyma) induced a significant decrease in the redox potential from 0.63 V^45^ (corresponding to the homoleptic complex) to 0.089 V, which agreed with its comparatively high HOMO energy. Based on the electronic features of the functional groups conjugated in *para*-positions, a higher HOMO energy (Figure 4a: **1** (−OMe) > **2** (−CH_3_) > **3** (−Cl)) involved a lower redox potential (Figure 4a). The experimental data showed good agreement with the calculated *E*_1/2_ values, as illustrated in Figure 4a. Additionally, we calculated the Mulliken charges and generated the corresponding HOMO surface density plots for complexes **1**–**4** and [Os­(bpy)_3_]^2+^ (Figure 4b and Figure S15, SI). Analyzing the **1–3** series, these results further confirmed that electron-withdrawing substituents reduced the electron density on the heavy atom, as indicated by the highest Mulliken charge for Os^2+^ in complex **3** (orange dots in Figure S15, SI) and the lowest for the chloride units on ligands dclb1 and dclb2 for the same complex (purple and blue dots in Figure S15, SI). On the other hand, complex **4**, bearing an anionic electron-donating carbometalated ligand, exhibited the greatest electron density at the metal center, and thus the lowest Mulliken charge for Os^2+^, compared to the non-carbometalated analogs. Since complexes **2** and **4** share the same bpy ancillary ligands (dmb), a comparison can be drawn to assess the effect of the carbometalated ligand. The carbometalated Mulliken charge (considering only the benzene moiety) is significantly higher than that of chloride (green dots in Figure S15, SI); in other words, the carbometalated moiety acts as a much stronger donor toward the metal center, as expected, decreasing *t*_2g_^π^ (metal) → π*­(ligand) back-donation.^64^ Consequently, the nearest methyl group (dmb 1, purple dots, see Figure S15, SI) retains more electron density, resulting in a lower Mulliken charge than in complex **2**. Following the same reasoning, the pyridine ligand (ampy) in complex **2**, positioned equivalently to the pyridine moiety in complex **4**, lies closer to the methyl group of dmb2, and thus shares essentially the same Mulliken charges (blue dots, Figure S15, SI). Since the pyridine moiety also behaves as a stronger electron donor toward Os­(II) compared to the chloride,^65^ this can further explain the charge differences between the two equivalent ancillary ligands in the **1–3** series: the chloride ligand is closer to dmb1/dmob1/dclb1, whereas the pyridine ligand is closer to dmb2/dmob2/dclb2 (purple and blue dots, respectively, see Figure S15, SI). Consequently, the Mulliken charge on these group units is consistently lower (meaning higher electron density) than that on the former ones (meaning lower electron density). Finally, a Hammett plot showing the correlation between the potential redox and the Hammett constants (σ) of the *para*-substituents on the bipyridine ligand was obtained (Figure S16, SI).^66^ As expected, σ and *E*_1/2_ or *E*_1/2_^calc^ displayed a positive linear correlation (R^2^ = 0.88 and 0.94, respectively).

**Figure 4:**
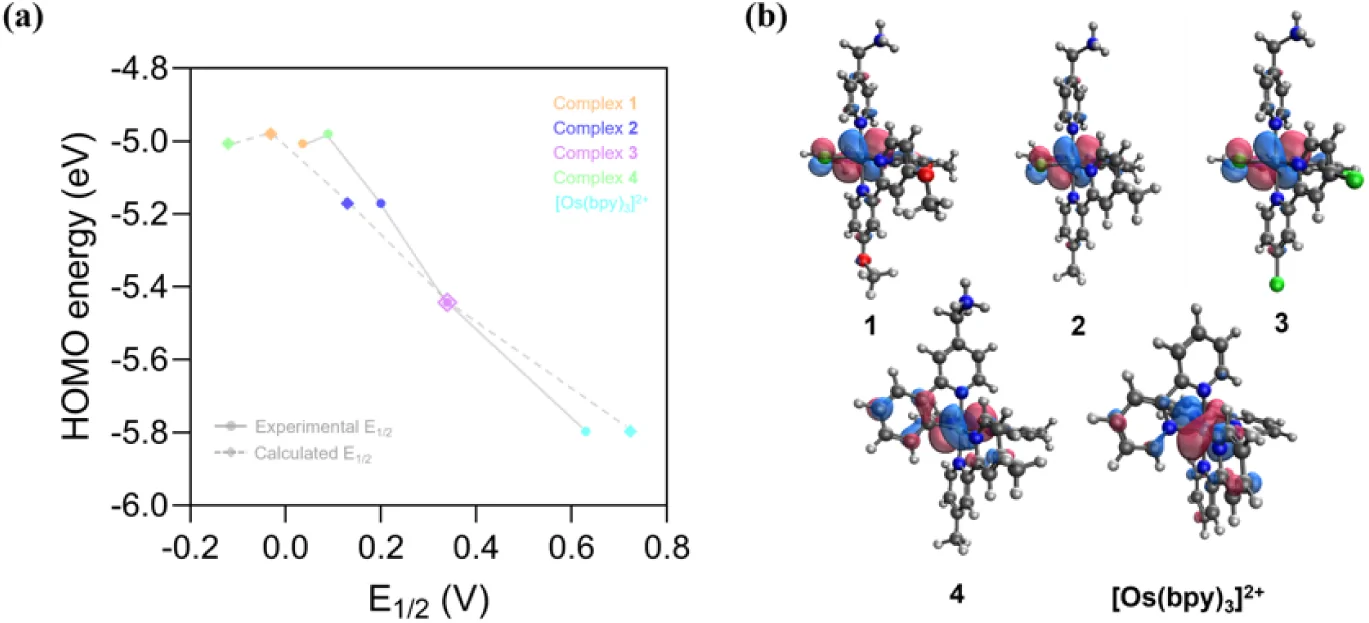
(a) HOMO energies and *E_1/2_* for complexes **1**–**4** and the reference [Os­(bpy)_3_]^2+^. (b) HOMO surface density plots for complexes **1**–**4**, and [Os­(bpy)_3_]^2+^. Positive/negative phases are shown in blue/red.

### 3.4 Covalent Attachment of Os­(II) Complexes to MUA/MCH Monolayers on Electrodes

To mimic the structure of a sensing SAM at the electrode interface and evaluate the efficiency of the covalent coupling method, complexes **1**–**4** were grafted onto mixed MUA/MCH monolayers on gold electrodes. This architecture was used as a simplified model interface to assess covalent coupling, electroactive reporter coverage, and retention of the reporter electrochemical properties after immobilization. In this system, MUA bears carboxylic acid groups, allowing covalent attachment via amide formation, while MCH acts as a backfiller to improve monolayer packing and reduce nonspecific interactions (see Figure S17, SI). Although this model interface does not constitute a complete EAB sensor, it reproduces key chemical features required for future integration of these reporters into thiol-based sensing architectures. As a control, we used NMB, a commercially available analog of MB, showing the same electrochemical properties but bearing two secondary amines for the coupling reaction.^30^ Electrochemical characterization of the modified electrodes was performed using both CV and SWV. To verify that the synthesized redox probes were covalently attached to the monolayer, rather than adsorbed nonspecifically onto the electrode surface, the coupling reaction was performed both in the presence and absence of EDC. Electrodes prepared with EDC displayed a clear, reversible redox signal in the cyclic voltammograms, whereas those prepared without EDC showed no redox peaks (Figure 5a and Figure S18, SI). This result confirmed the formation of covalent bonds between the redox probes and the MUA/MCH mixed monolayer. Corresponding square wave voltammograms are shown in Figure 5b. The traces in Figure 5b are normalized to their peak currents to facilitate comparison of potential positions and should not be interpreted as evidence for identical coupling efficiencies. Raw peak currents (*I*_*p*_) are provided in Table S2 in the SI, where they show reporter-dependent immobilization and/or interfacial response. Despite these differences in absolute signal amplitude the peak positions demonstrate systematic potential modulation across complexes **1**–**4** upon surface immobilization as a function of the ligand electronic nature. This behavior was consistent with the ligand-electronics trends previously observed in solution, with the peak positions shifting toward less positive potentials in the sequence −Cl (complex **3**), −Me (complex **2**), carbometalated ppyma (complex **4**), and −OMe (complex **1**). Notably, the formal redox potentials of all Os­(II)-based redox reporters proposed in this work fell within the narrow potential window optimal for electrochemical sensing applications, in contrast to the gold-standard NMB (Figure 5b).

**Figure 5:**
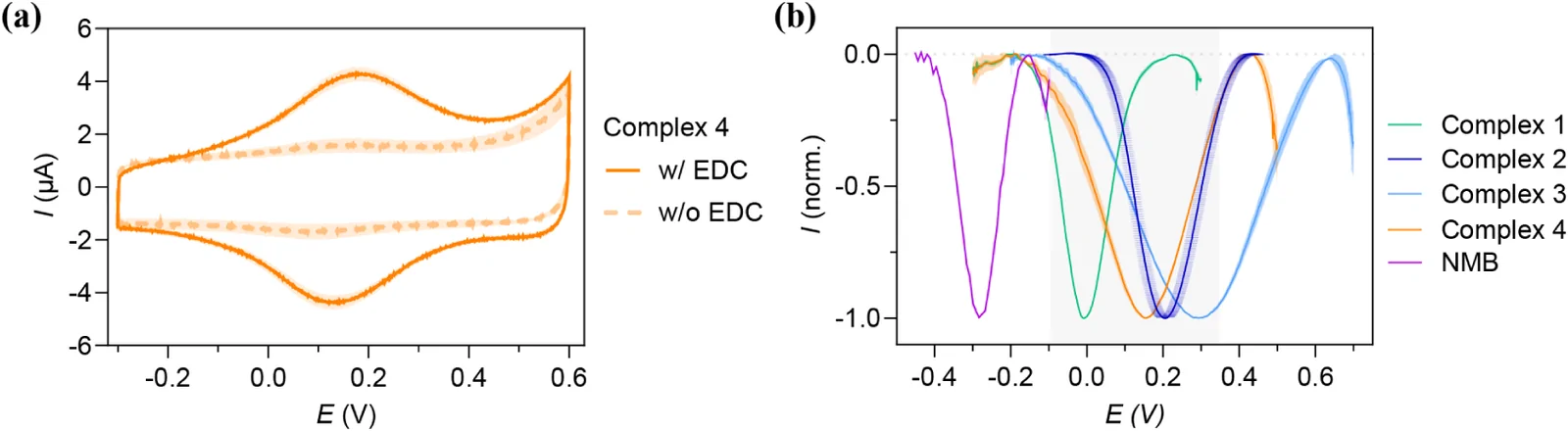
(a) Cyclic voltammograms in PBS (pH 7.4) for complex **4** after the coupling reaction in the presence (solid line) and absence (dashed line) of EDC (n = 3). Measurements were collected using a scan rate of 5 V s^–1^, and a voltage step of 1 mV. (b) Normalized square wave voltammograms in PBS (7.4) for complexes **1–4** attached to MUA monolayers on gold electrodes (n = 3). The shadowed area highlights the optimal potential window for electrochemical sensing. All measurements were collected using a pulse amplitude of 25 mV, a voltage step of 1 mV, and a frequency of 500 Hz or, in the case of NMB, a voltage step of 8 mV and a frequency of 25 Hz.

The electroactive surface coverage (*Γ*_*redox*_) of each immobilized Os­(II) reporter was calculated from the integrated charge of the background-corrected surface CVs using *Γ*_*redox*_ *= Q/nFA*, with n = 1 for the Os­(II/III) couples. The resulting *Γ*_*redox*_ values were 2.46 ± 1.30, 16.43 ± 8.42, 4.50 ± 1.99, and 14.36 ± 1.09 pmol cm^–2^ for complexes **1**–**4**, respectively (Table S2), indicating reporter-dependent immobilization and electrochemical accessibility. Therefore, differences in absolute SWV current among the immobilized complexes cannot be interpreted solely as differences in redox potential.

In parallel, the total thiolated SAM density (*Γ*_*thiol*_) was quantified by reductive desorption in 0.1 mol L^—1^ KOH, as described in detail in the SI. The *Γ*_*thiol*_ values for the Os-modified electrodes were in the range of 1.7–2.3 nmol cm^–2^, comparable to the MUA-only reference surface, confirming that thiolated monolayers were formed in all cases with similar overall surface densities (Table S3). Thus, the differences in *Γ*_*redox*_ likely arise from reporter-dependent coupling efficiency rather than from large differences in SAM formation. Comparison of *Γ*_*redox*_ with the absolute SWV peak currents shows that surface coverage alone does not fully explain the faradaic response. For instance, complexes **2** and **4** showed relatively high electroactive coverages (16.4 and 14.4 pmol cm^—2^, respectively), but complex **2** produced a much larger SWV current at 500 Hz. This suggests that molecular orientation, electronic coupling, and apparent interfacial electron-transfer efficiency also influence the observed signal. The *|Ip|/Γ*_*redox*_ ratio is therefore reported as an operational measure of the SWV response per electroactive reporter, but not as a formal heterogeneous electron-transfer rate constant.

### 3.5 Electrochemical and pH Stability of Os­(II)-Modified SAMs

One of the key advantages of using these metal complexes for electrochemical sensing applications is their pH-independent redox behavior, which potentially enables biosensing in pH-variable media such as sweat (pH 5.0–7.5), urine (4.5–8.0), or even blood (pH 6.9–7.6 in cases of acidosis or alkalosis disorders). To confirm pH robustness, we evaluated the electrochemical response by SWV in McIlvaine buffer over acidic to basic conditions. For all four Os­(II) complexes, no measurable peak potential deviation was observed across the different pH conditions, and the peak shapes remained essentially unchanged (Figure 6a-d). This demonstrated that the Os­(II/III) couple is effectively pH-independent when immobilized on MUA/MCH monolayers. A slight variation was observed for complex **4** at the most acidic condition (pH 4.5), manifested as a slight peak shift and current loss (Figure 6d). This could be attributed to a proton incursion in the carbometalated arm,^67^ which may weaken the C–Os bond and accelerate demetallation. By contrast, the benchmark NMB exhibited a strong pH-dependency (Figure 6e). This expected behavior arises from the proton exchange involved in its redox process, leading to formal potential shifts of up to 0.2 V. Thus, the pH-insensitive redox signal of these Os­(II) complexes makes them well-suited for sensing in matrices with fluctuating or poorly controlled pH and an alternative to the gold standard MB.

**Figure 6:**
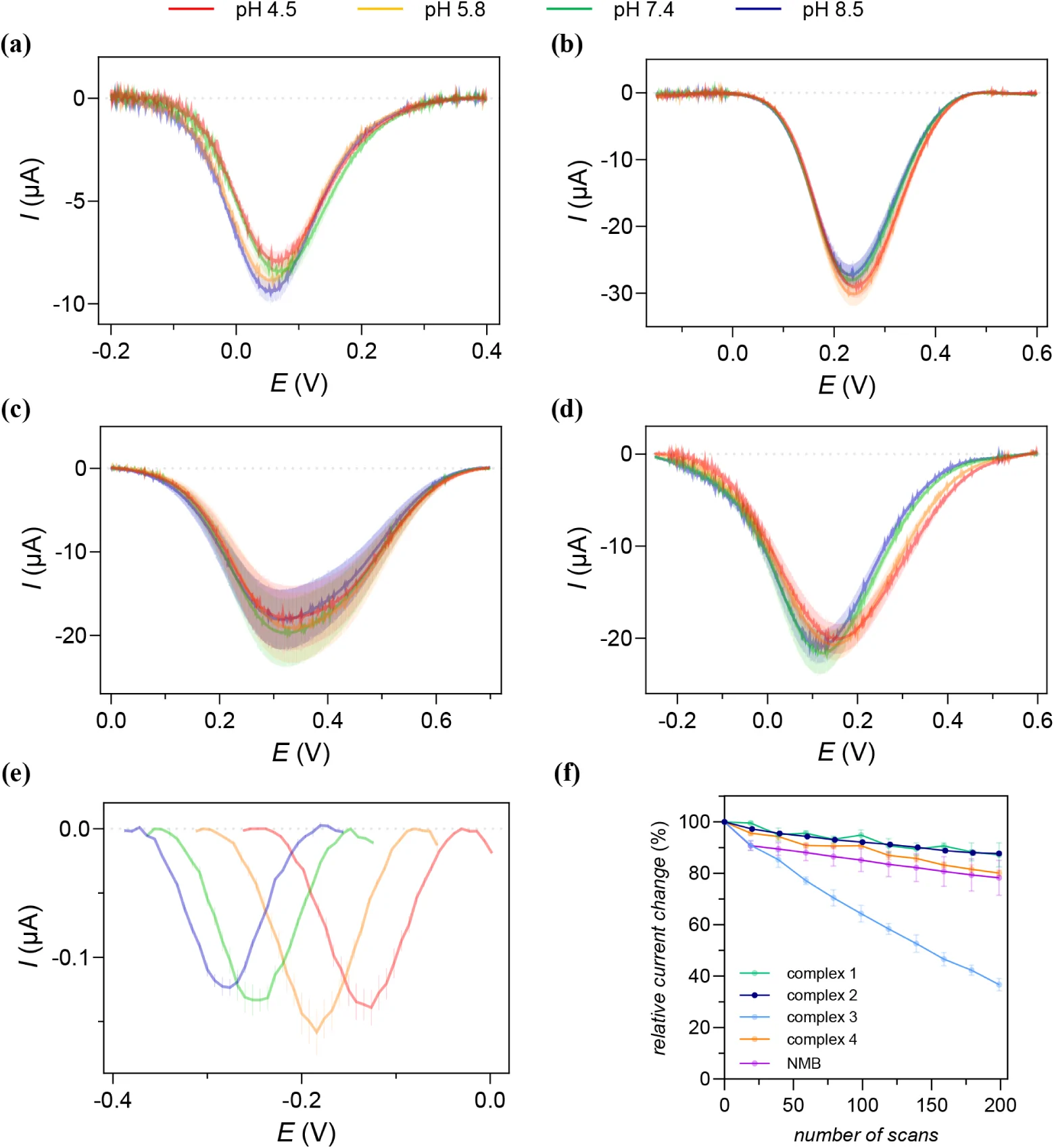
Square wave voltammograms for (a) complex **1**, (b) complex **2**, (c) complex **3**, (d) complex **4**, and (e) NMB attached to mixed MUA/MCH monolayers on gold electrodes, recorded in McIlvaine buffer at variable pH. All measurements were collected using a pulse amplitude of 25 mV, a voltage step of 1 mV, and a frequency of 500 Hz or, in the case of NMB, a voltage step of 8 mV and a frequency of 25 Hz (n = 3). (f) Relative current change in peak current over 200 SWV scans obtained in McIlvaine buffer using Au electrodes with complexes **1**–**4** and NMB immobilized to linear carbon chains via amide bond formation (n = 3). For clarity, only the relative current change values recorded every 20 scans are displayed. For this experiment, all measurements were collected using a pulse amplitude of 25 mV, a voltage step of 1 mV, and a frequency of 500 Hz.

Finally, we evaluated the operational stability of the surface-immobilized reporters under repeated square wave interrogation (Figure 6f). Complexes **1**, **2**, and **4** exhibited stable peak currents over 200 consecutive scans (<20% current decrease), whereas complex **3** and the gold standard NMB showed a 60% and 40% current loss, respectively, upon 200 SWV scans. The decrease in peak intensity observed during repeated SWV interrogation is attributed primarily to changes at the gold-SAM interface rather than to decomposition of the Os reporters. The SAM prepared with the previously reported Os­(II) complex^30^ (complex **2** here), and that prepared with complex **1** showed the highest stability among our Os­(II)-based reporters. Although Os­(II/III) polypyridyl complexes are generally chemically robust, the electrochemical signal of surface-confined reporters is highly dependent on the integrity and electronic coupling of the thiolated monolayer. Among the Os complexes, complex **3** operates at the most positive potential, close to the onset of anodic background processes associated with the gold surface under our experimental conditions (Figure S19). Repeated interrogation in this potential region may perturb the Au–thiol/SAM interface, contributing to the larger signal loss observed for this reporter. In contrast, complexes **1**, **2**, and **4** operate at less positive potentials and show improved signal retention. For NMB, the interrogation window overlaps more strongly with negative-potential background processes, including oxygen reduction (Figure S19), which may also contribute to signal degradation through changes in the local interfacial environment.

The latter demonstrated the promising use of these reporters in electrochemical biosensing. Although the toxicity of the complexes reported herein has not been evaluated, similar structure-related complexes demonstrated low dark toxicity.^58^ Future studies will be required to evaluate the biocompatibility and toxicity of these complexes for specific biosensing applications.

## 4 Conclusions

We developed a family of polypyridyl Os­(II) complexes as redox reporters designed to address two persistent limitations of gold-standard probes (e.g., MB): constrained potential tunability and pH-coupled redox behavior. A thorough spectroscopic, electrochemical, and computational study allowed us to define how electronic and structural factors modulate the redox properties of these reporters. By varying the ancillary ligands, the metal-based oxidation potential of the Os­(II) complexes was systematically tuned across a sensing-friendly window (0.04 to +0.34 V *vs* Ag/AgCl), while retaining fast, quasi-reversible one-electron behavior. A key finding was that substitution of one Os-N bond with either an Os-C or Os-Cl bond led to a drastic decrease in the oxidation potential induced by an increase in the HOMO levels energy in comparison with their homoleptic analogs. Computational modeling reproduced the experimental results, linking the cathodic/anodic shifts to systematic changes in HOMO energy and metal–ligand charge distribution and establishing practical design rules for redox tuning. Their oxidation potential, positioned within the aqueous “*sweet spot*” (≈−0.1 to +0.4 V *vs* Ag/AgCl), underscored their strong potential for the development of electrochemical sensors. To validate biosensor compatibility, we demonstrated that our complexes can be covalently immobilized on gold substrates via amide coupling to carboxylate-terminated MUA/MCH monolayers while preserving their electrochemical features. Critically, the immobilized Os­(II) redox probes exhibited pH-independent peak potentials across acidic to basic conditions, in stark contrast to the large potential shifts observed for NMB. Operational stability under rapid and continuous square-wave interrogation was observed for complexes **1**, **2**, and **4**, whereas complex **3** and NMB showed substantial signal loss, consistent with the hypothesis that scanning outside the optimal electrochemical window accelerates SAM degradation. This highlights the importance of redox modulation for developing electrochemical sensors. This study paves the way for the design of new redox probes with on-demand potentials and improved cycling stability compared to the current gold standard, MB. These attributes are expected to be particularly advantageous for EAB sensing and for advanced formats that benefit from well-separated redox signatures (e.g., ratiometric and multiplexed sensing).

## Supplementary Material



## Notes

1.General schemes of the synthesis of Os­(II) complexes and electrodes preparation, mass spectra for all compounds, and additional spectroscopic and electrochemical details (PDF)

## Abbreviations

ampy: 4-(aminomethyl)­pyridine
CV: cyclic voltammetry
DCM: dichloromethane
dclb: 4,4’-dichloro-2,2’-bipyridine
DI: deionized
dmb: 4,4’-dimethyl-2,2’-bipyridine
DMF: *N*,*N*-dimethylformamide
dmob: 4,4’-dimethoxy-2,2’-bipyridine
${E_{0}}^{\prime }$: formal potential
E_1/2_: half-wave potential
EAB: electrochemical aptamer-based
EDC: 1-ethyl-3-(3-dimethylaminopropyl)­carbodiimide
EtOH: ethanol
GC: guanidinium hydrochloride
HOMO: highest occupied molecular orbital
LLCT: ligand-to-ligand charge transfer
MB: methylene blue
MCH: 6-mercapto-1-hexanol
MeOH: methanol
MLCT: metal-to-ligand charge transfer
MUA: 11-mercaptoundecanoic acid
NMB: new methylene blue
PBS: phosphate buffer saline
ppyma: (2-phenylpyridin-4-yl)­methanamine
SAM: self-assembled monolayer
SWV: square wave voltammetry
TD-DFT: time dependent density functional theory.
