# Supplementary material for: Tunable Osmium Complexes as pH-Insensitive Redox Reporters for Electrochemical Biosensing
Source: Langmuir. 2026 Aug 12;42(33):24074–85. doi: 10.1021/acs.langmuir.6c02628 (PMC13523742; doi:10.1021/acs.langmuir.6c02628)
Supplement: Supplementary file 1 [file la6c02628_si_001.pdf]

Tunable Osmium Complexes as pH-Insensitive Redox Reporters for Electrochemical Biosensing

Andrea Montón−Vicente[1,2,3], Jose Quílez−Alburquerque[1,], Lorenzo Gramolini[4], Lucía Morillo−Victorero[1,5], Cristina García−Iriepa[4,6], Marco Marazzi[4,6], David Díaz Díaz[2,3,*], and María Alba[1,*]*

[1]ARQUIMEA Research Center, San Cristóbal de La Laguna 38320, Spain

[2]AFM-NANO, Instituto Universitario de Bio-Orgánica Antonio González (IUBO-AG), Universidad de La Laguna, La Laguna 38206, Spain

[3]Departamento de Química Orgánica, Universidad de La Laguna, La Laguna 38206, Spain

[4]Universidad de Alcalá, Departamento de Química Analítica, Química Física e Ingeniería Química, Alcalá de Henares, Madrid 28805, Spain

[5]Institute of Chemical Research of Catalonia, Tarragona 43007, Spain

[6]Universidad de Alcalá, Instituto de Investigación Química "Andrés M. del Río" (IQAR), Alcalá de Henares, Madrid 28805, Spain

*Correspondence: jquilez@arquimea.com; ddiazdiaz@ull.edu.es; malba@arquimea.com

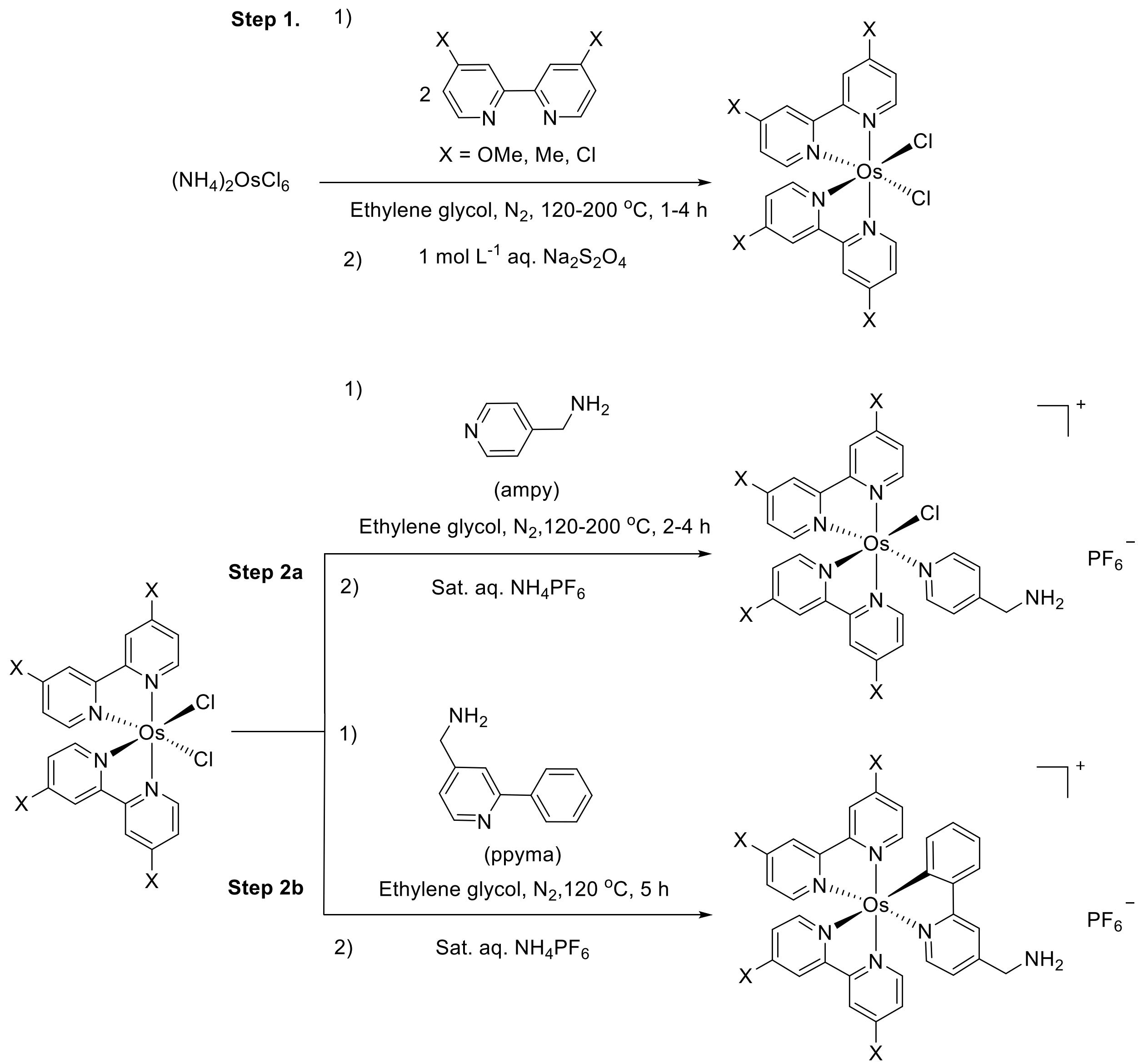

Scheme S1. General scheme of the two-step synthetic route to the Os(II) complexes studied in this work, involving preparation of the *cis*-[Os(NN)$_2$Cl$_2$] precursor and subsequent ligand substitution to afford complexes **1**–**4**.

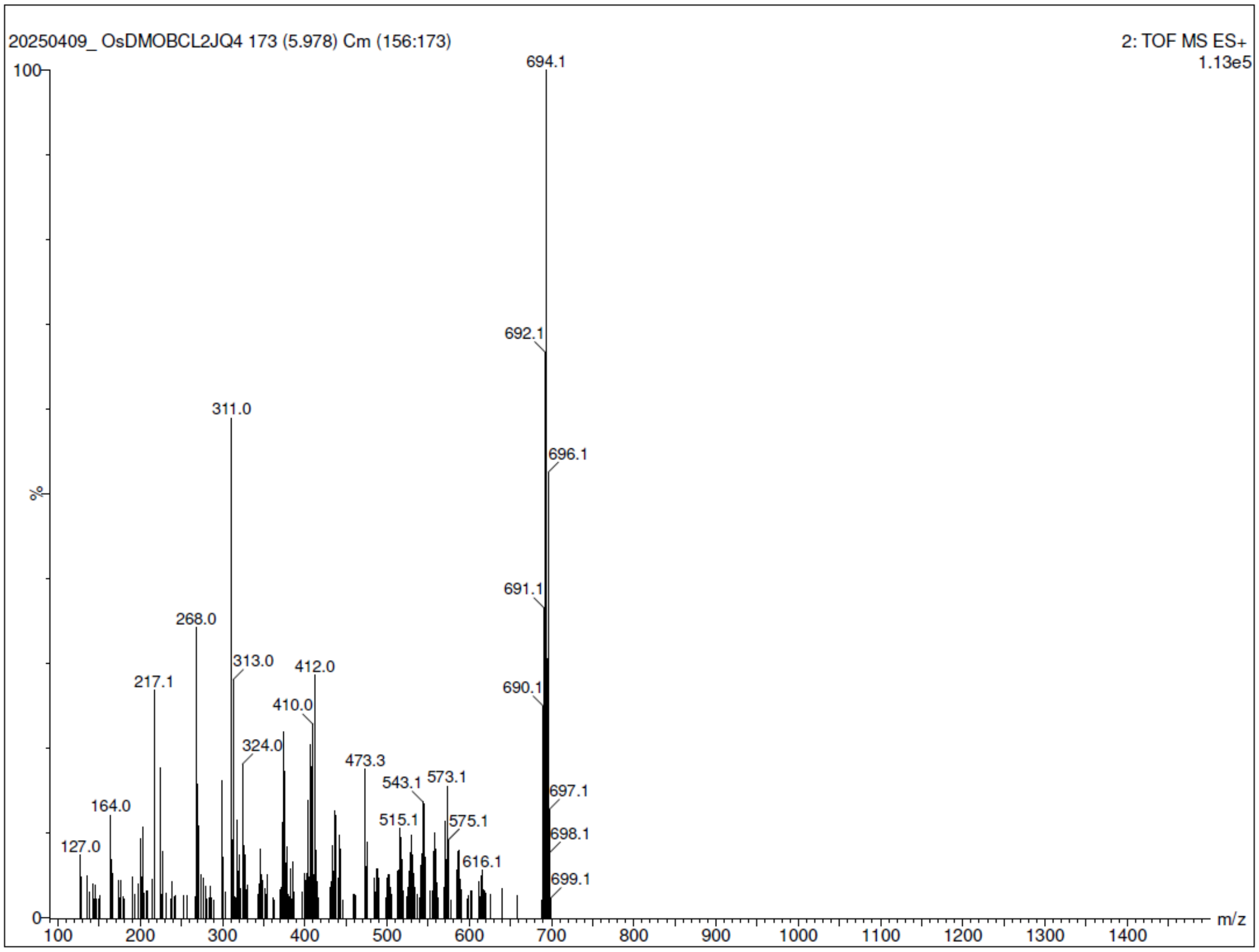

Figure S1. Mass spectrum of [Os(dmob)$_2$Cl$_2$] by electrospray ionization in positive detection mode; m/z: [M] calcd. for [$C_{24}H_{24}Cl_2N_4O_4Os$], 694.1; found, 694.1.

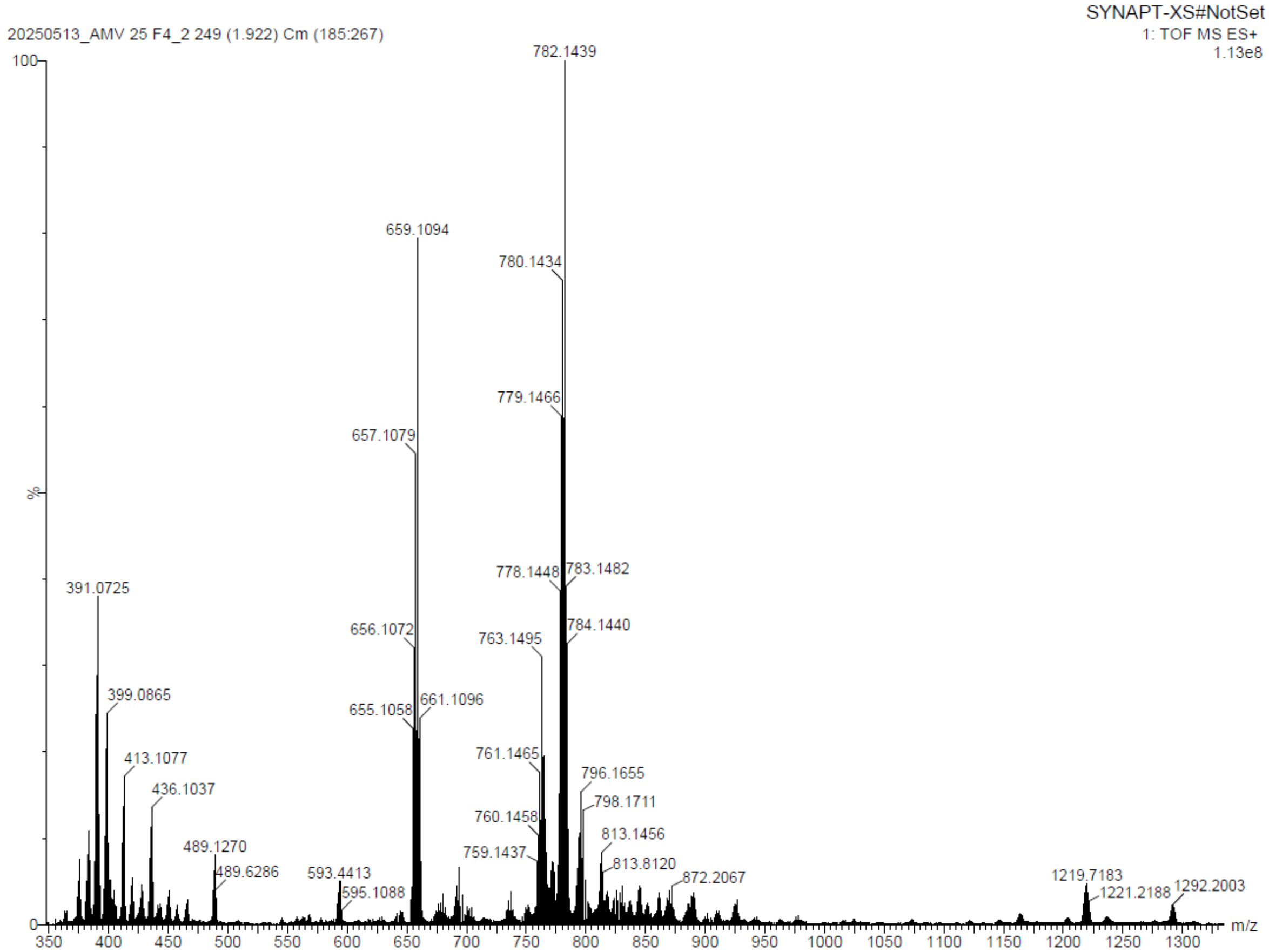

Figure S2. Mass spectrum of $[Os(dmob)_2(ampy)Cl]^{2+}$ (complex **1**) by electrospray ionization in positive detection mode; m/z: [M] calcd. for $[C_{30}H_{34}ClN_6O_5Os]^+$, 784.3; found, 782.1.

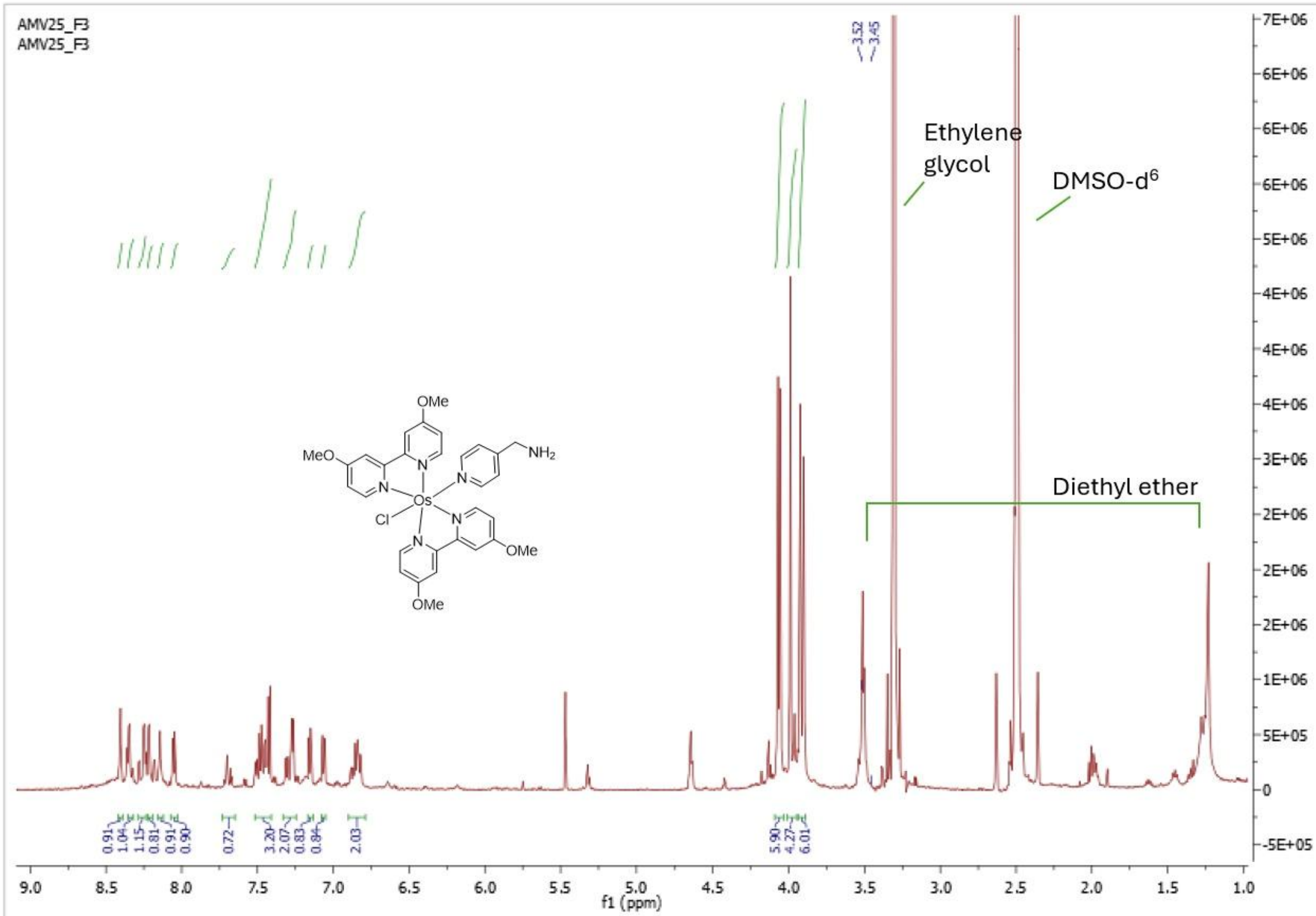

Figure S3. 500-MHz ^{1}H-NMR spectrum of complex **1** in DMSO-d6.

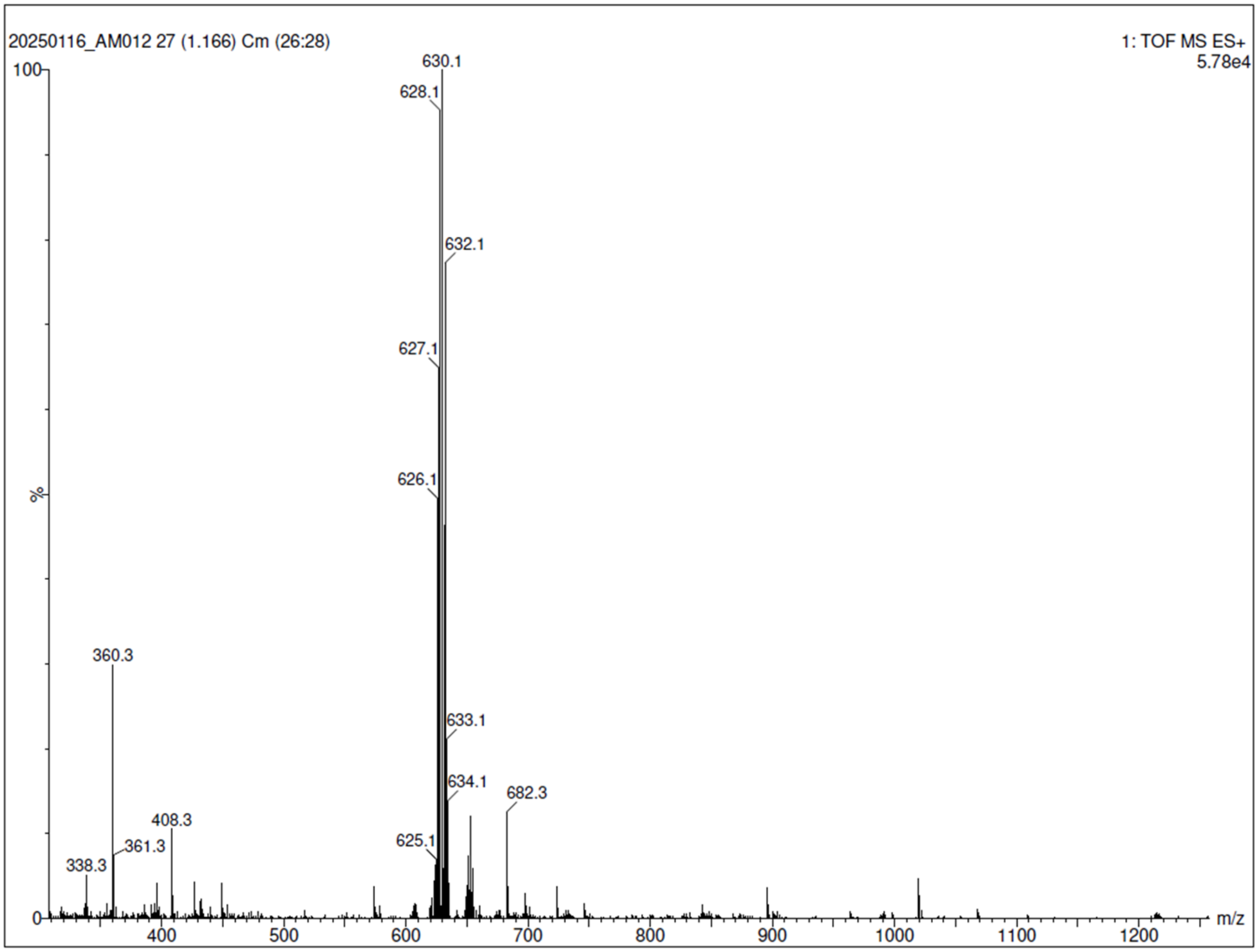

Figure S4. Mass spectrum of $[Os(dmb)_2Cl_2]$ by electrospray ionization in positive detection mode; m/z: [M] calcd. for $[C_{24}H_{24}Cl_2N_4Os]$, 630.1; found, 630.1.

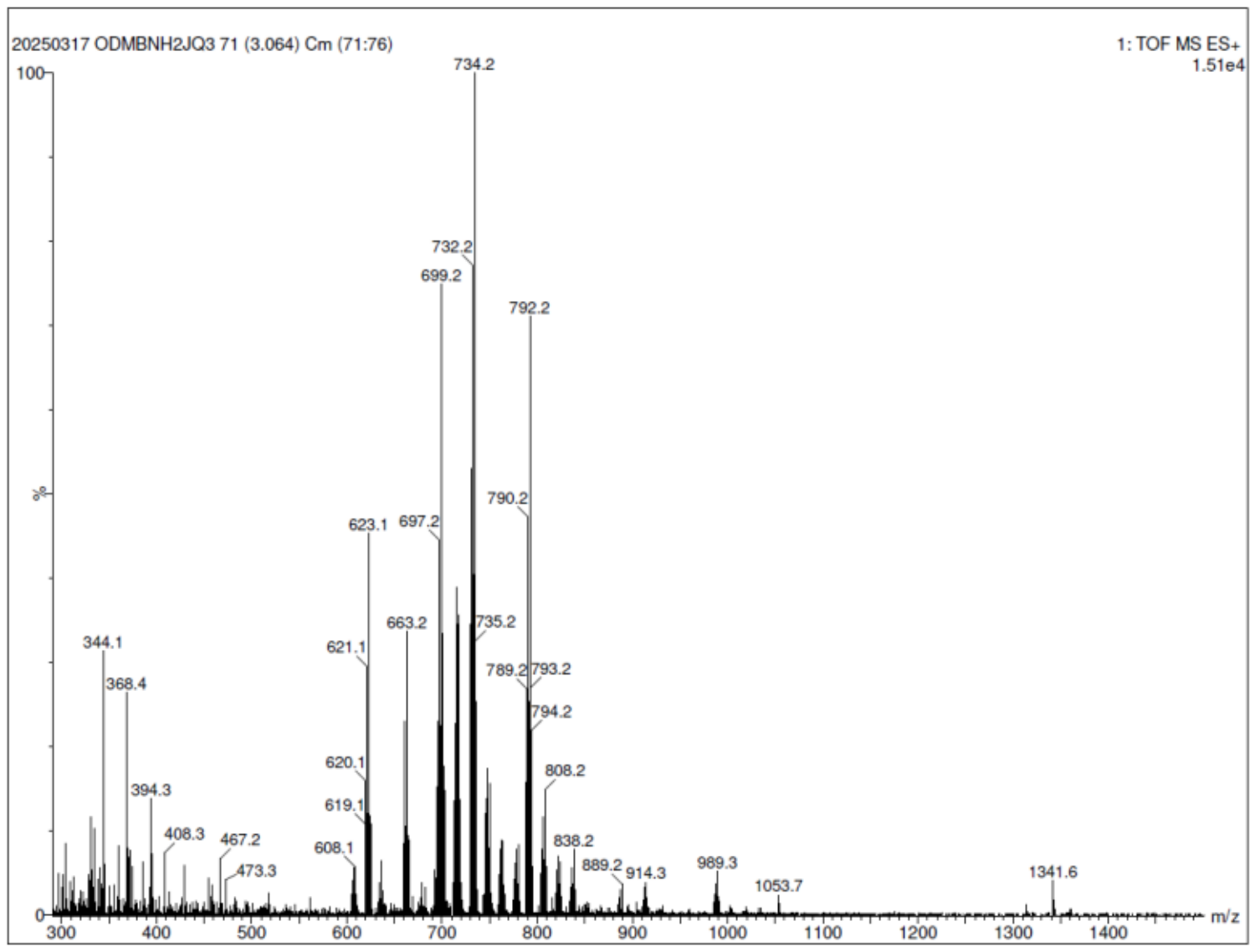

Figure S5. Mass spectrum of $[Os(dmb)_2(ampy)Cl]^{2+}$ (complex **2**) by electrospray ionization in positive detection mode; m/z: [M] calcd. for $[C_{30}H_{32}ClN_6Os]^+$, 703.2; found, 703.2.

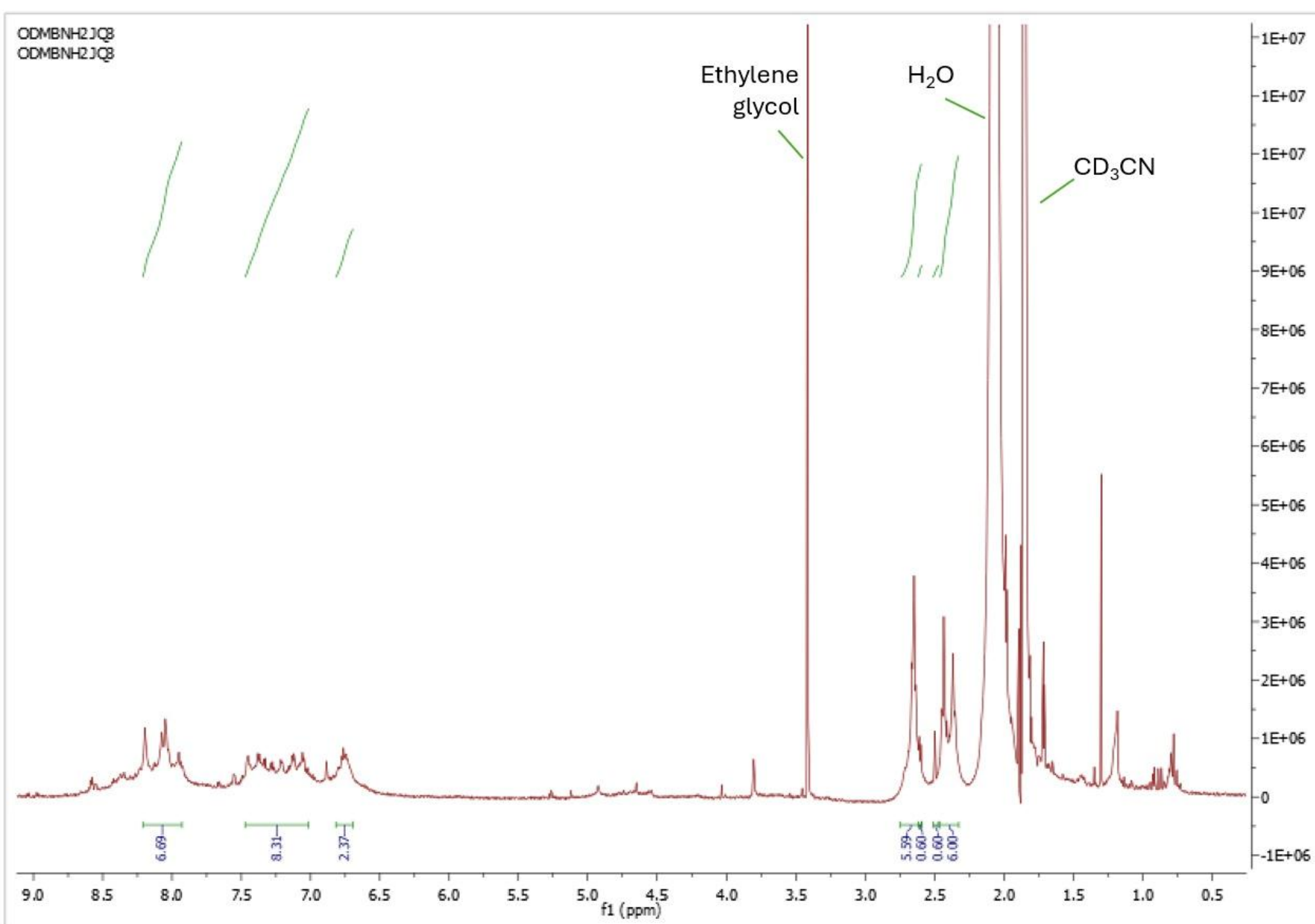

Figure S6. 500-MHz ^{1}H-NMR spectrum of complex **2** in acetonitrile-d3.

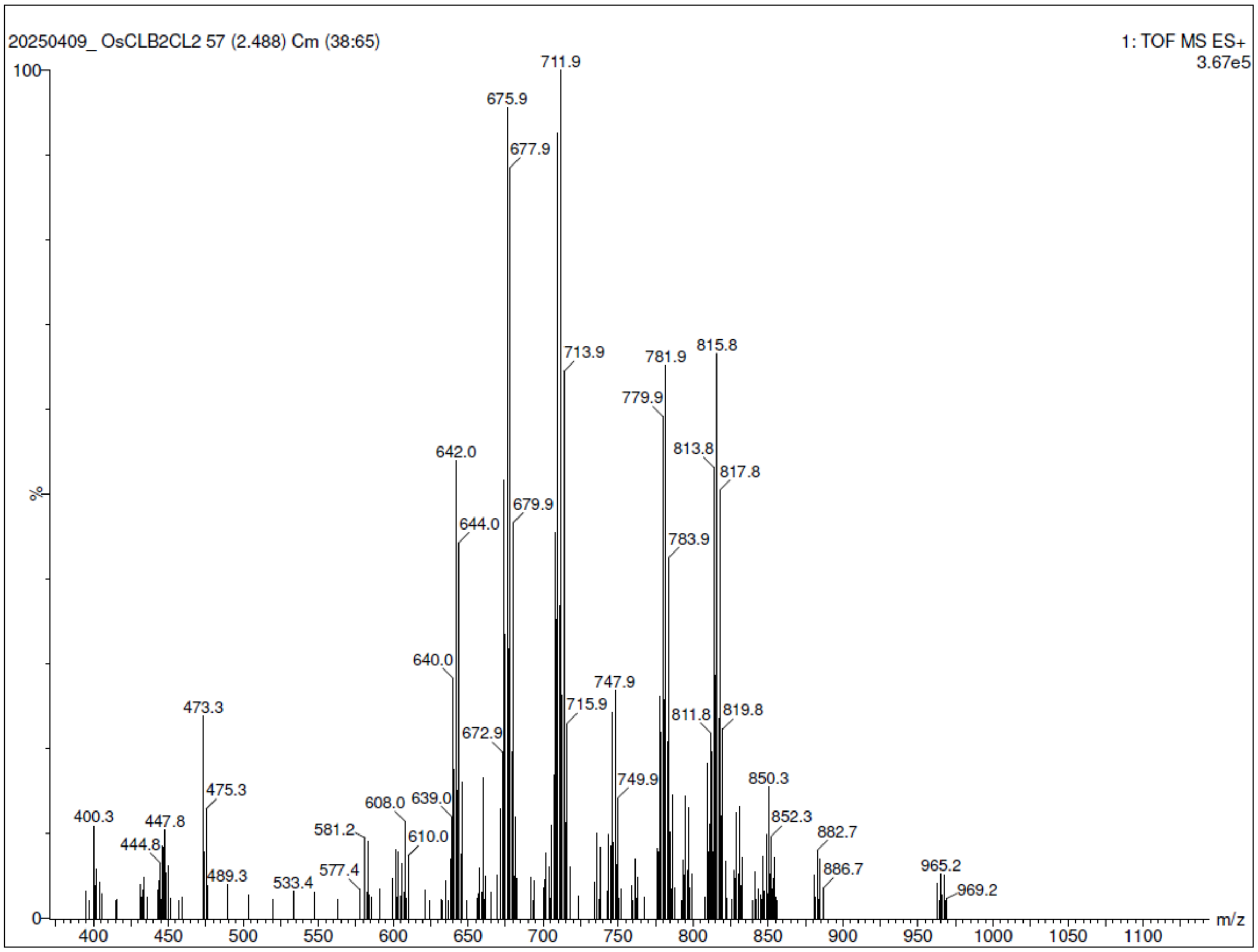

Figure S7. Mass spectrum of [Os(dclb)$_2$Cl$_2$] by electrospray ionization in positive detection mode; m/z: [M] calcd. for [$C_{20}H_{12}Cl_6N_4Os$], 711.3; found, 711.9; [$C_{20}H_{12}Cl_5N_4Os$], 675.0; found, 675.9.

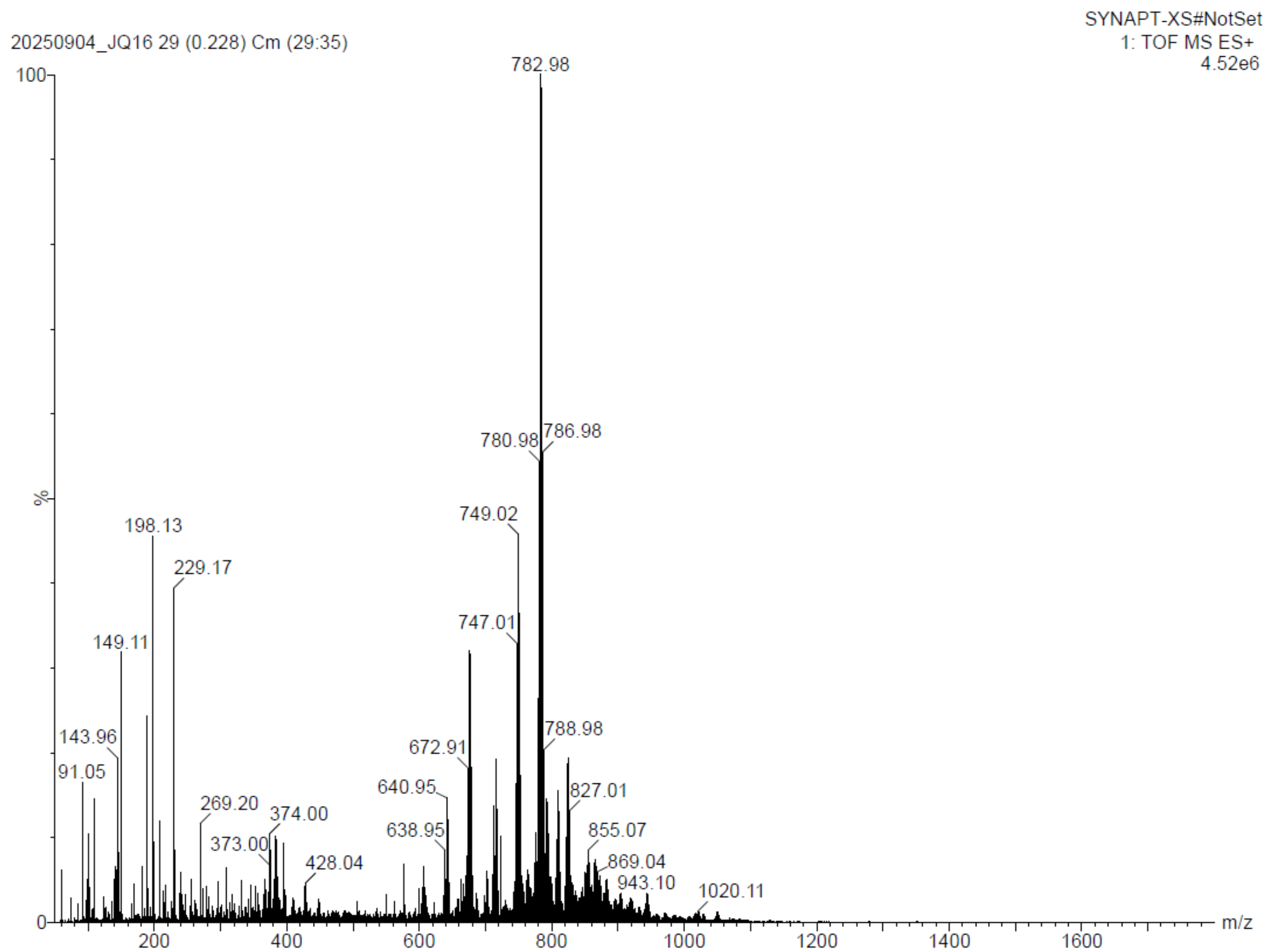

Figure S8. Mass spectrum of $[Os(dclb)_2(ampy)Cl]^{2+}$ (complex **3**) by MALDI detection mode; m/z: [M] calcd. for $[C_{26}H_{20}Cl_5N_6Os]$, 784.9; found, 782.9.

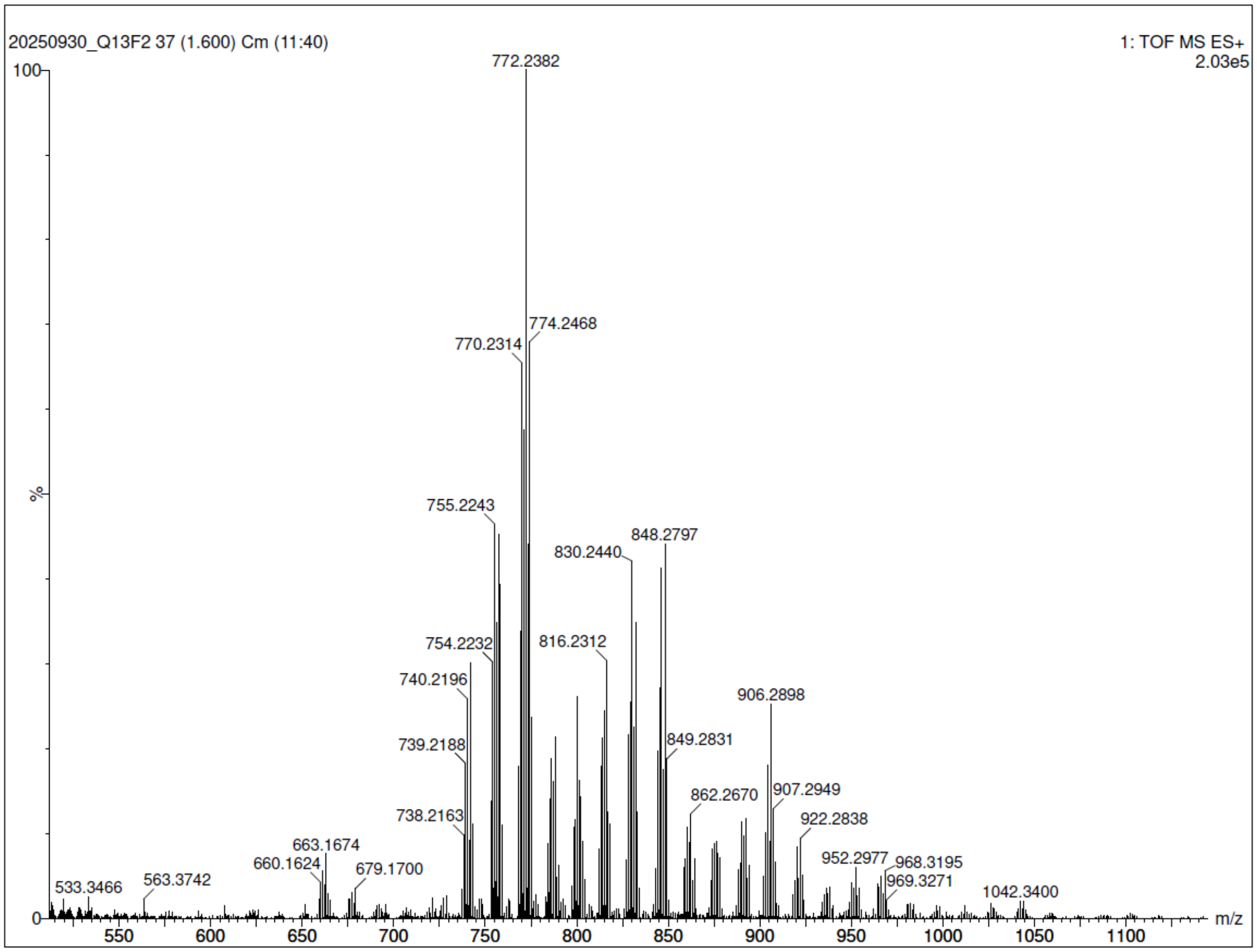

Figure S9. Mass spectrum of $[Os(dmb)_2(ppyma)]^{2+}$ (complex **4**) by electrospray ionization in positive detection mode; m/z: [M] calcd. for $[C_{37}H_{37}N_6OOs]$, 771.9; found, 772.2.

	***Trans* isomer**	***Cis* isomer**	
**Complex 1**	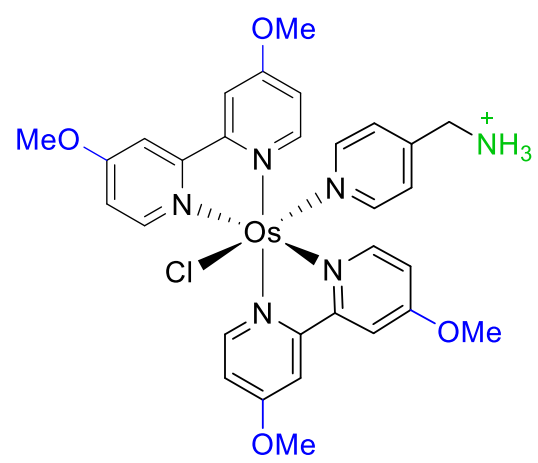	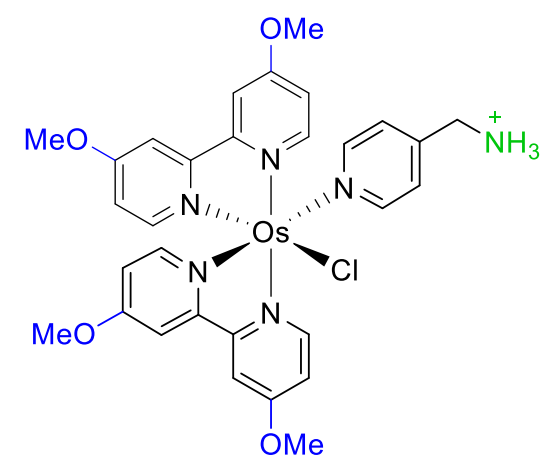	$\Delta G_{trans\text{-}cis}$ = 10.6 kcal mol^{-1}
**Complex 2**	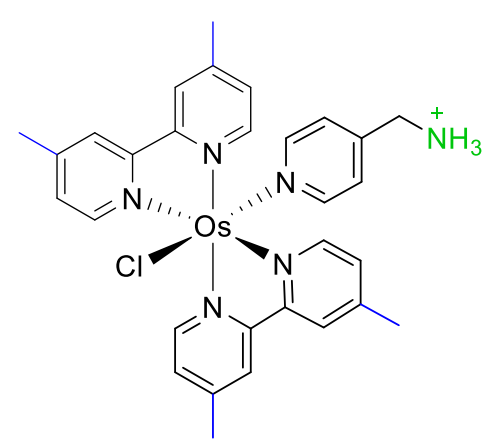	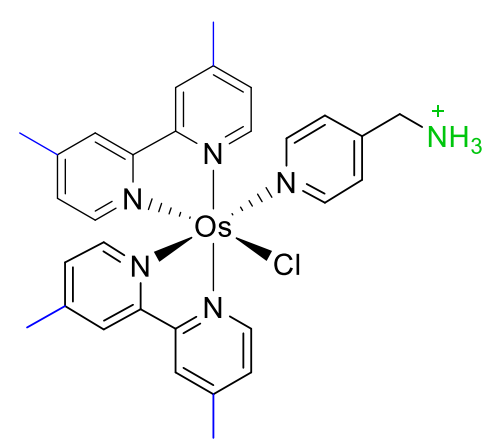	$\Delta G_{trans\text{-}cis}$ = 11.4 kcal mol^{-1}
**Complex 3**	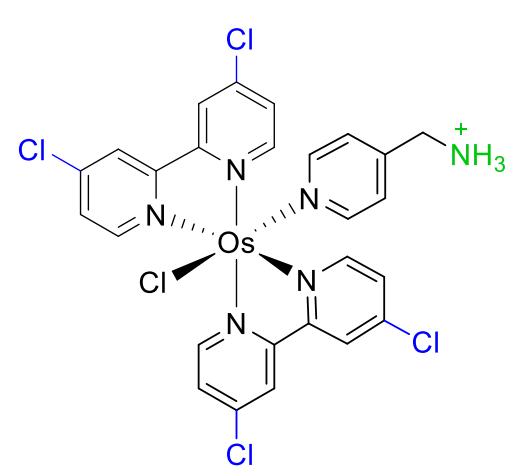	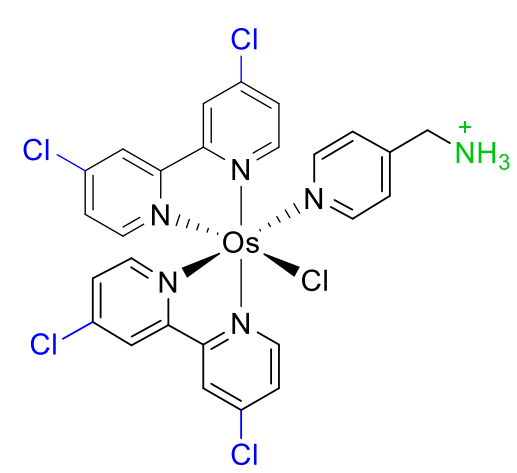	$\Delta G_{trans\text{-}cis}$ = 11.7 kcal mol^{-1}

Figure S10. Calculated *cis* vs *trans* free energies at 298.15 K (the thermal energy at 298.15 K is kbT=0.592 kcal mol^{-1}).

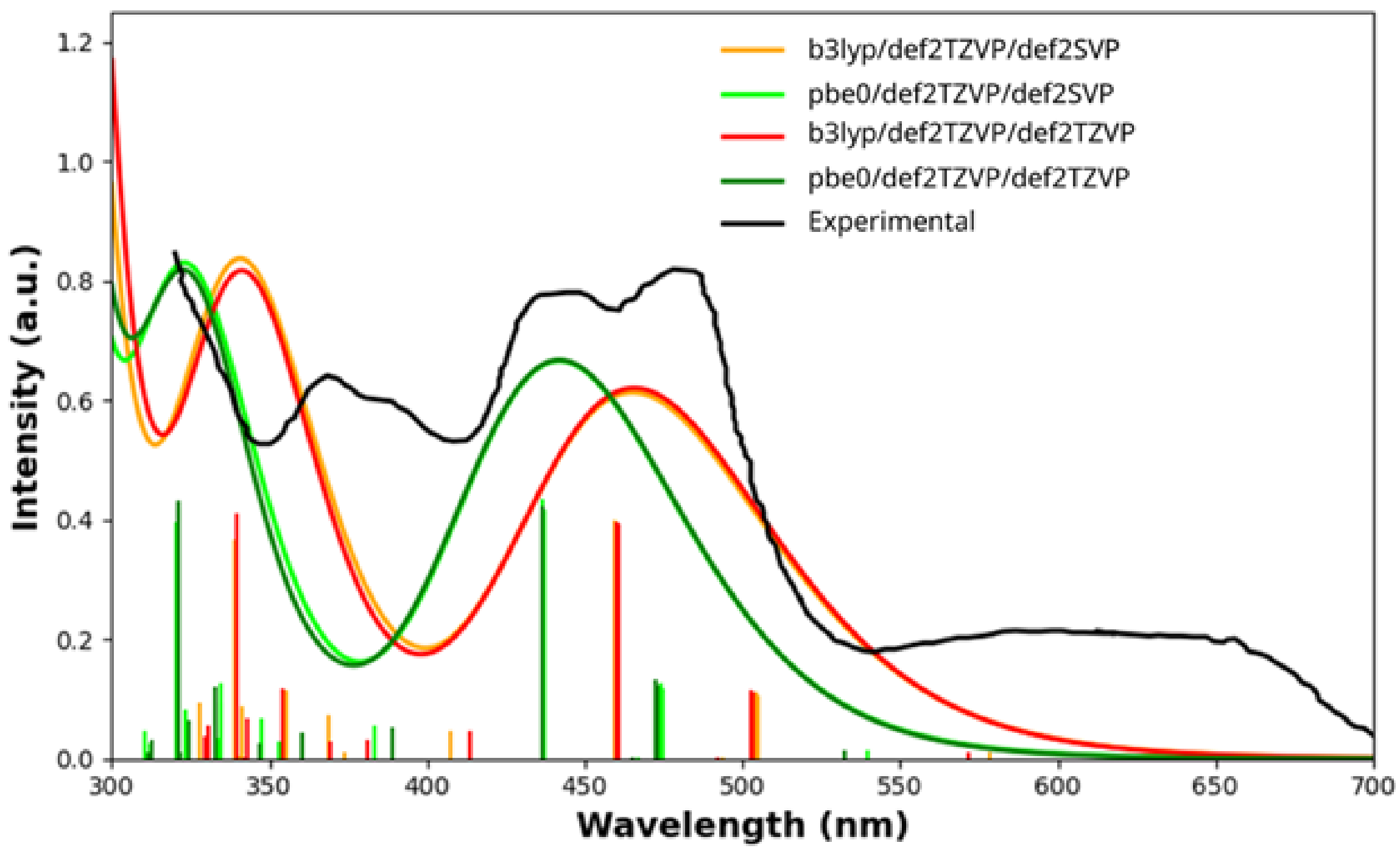

Figure S11. Absorption spectra of $[Os(bpy)_3]^{2+}$ calculated at different levels of theory (functional / Os basis set / non-metal basis set) in comparison with the experimental spectrum in acetonitrile[1], for benchmarking purposes. The calculations were performed with IEFPCM implicit solvation (acetonitrile).

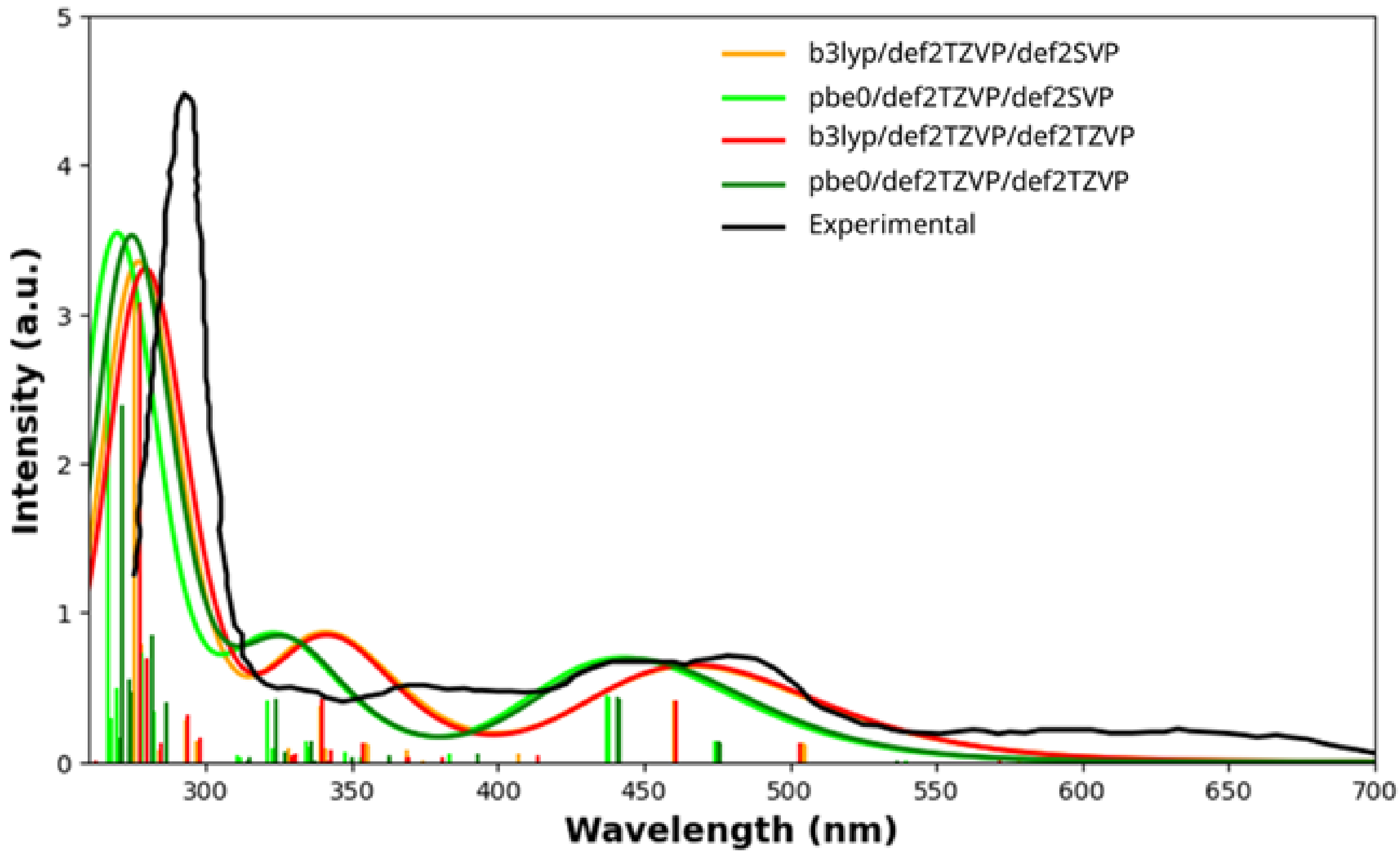

Figure S12. Absorption spectra of $[Os(bpy)_3]^{2+}$ calculated at different levels of theory (functional / Os basis set / non-metal basis set) in comparison with the experimental spectrum in DMF[2], for benchmarking purposes. The calculations were performed with IEFPCM implicit solvation (DMF).

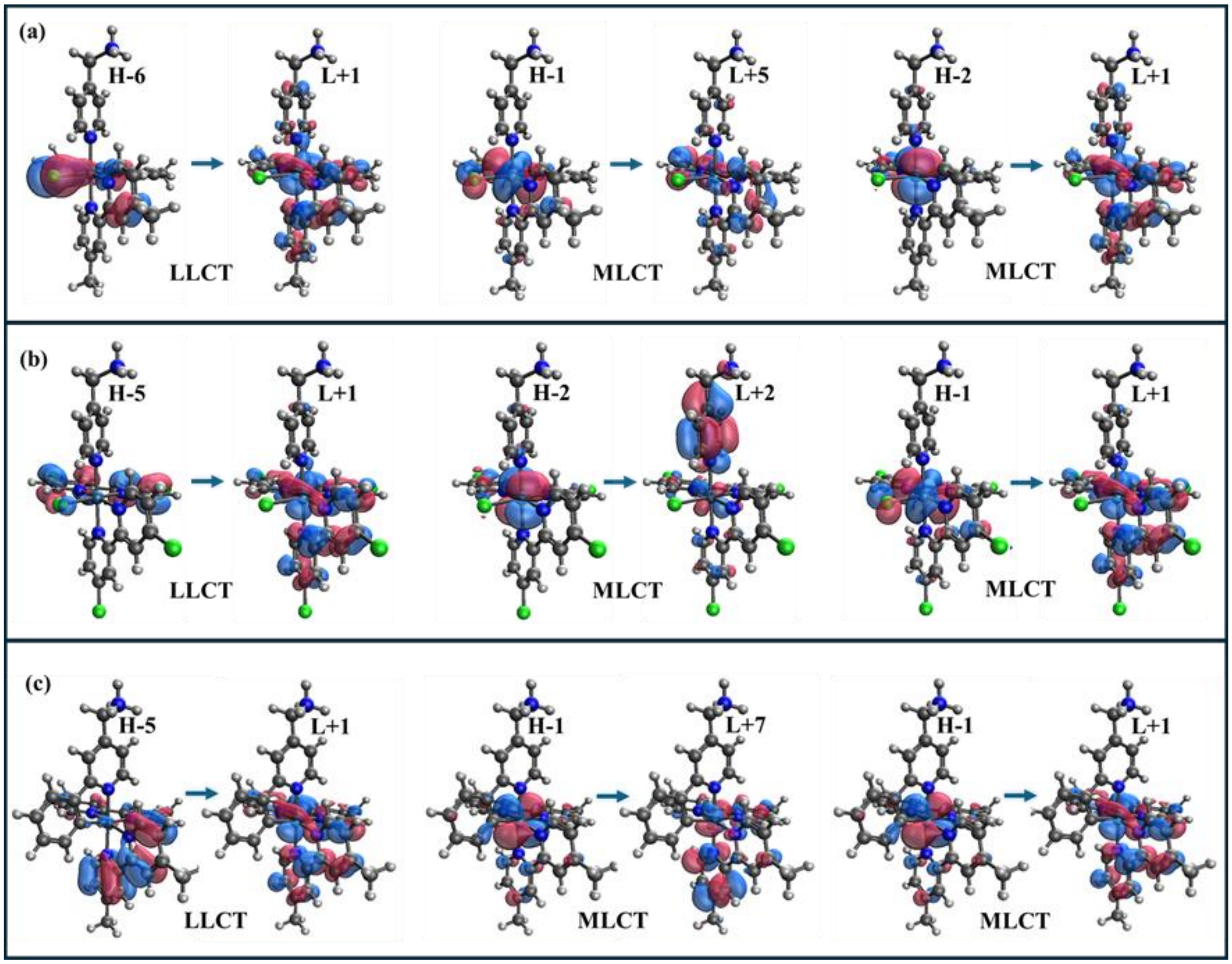

Figure S13. Orbitals involved in the main electronic transitions and their characters for a) complex **2** b) complex **3** and c) complex **4** (H and L denote HOMO and LUMO, respectively; LLCT=ligand-to-ligand-charge-transfer, MLCT=metal-to-ligand-charge-transfer).

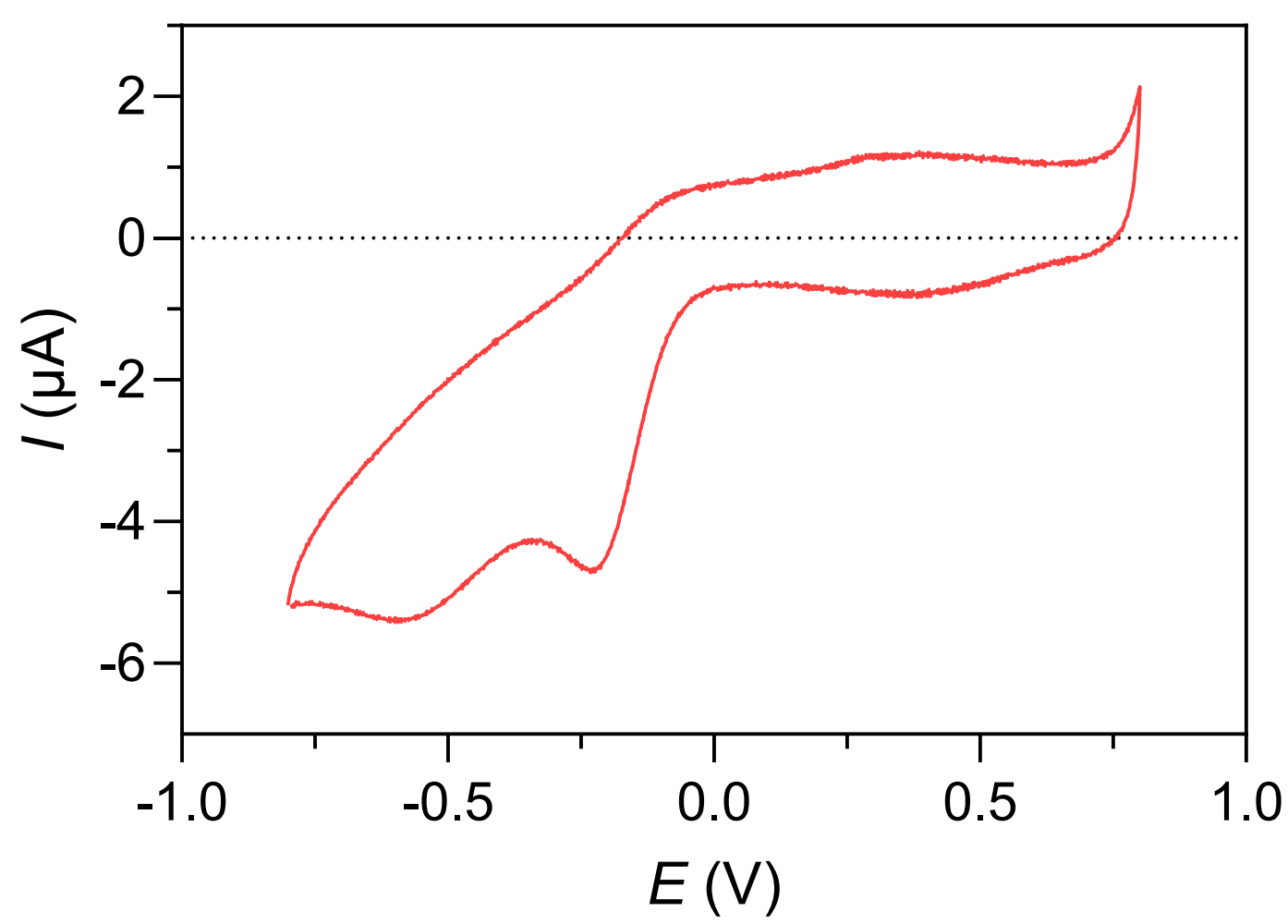

Figure S14. Cyclic voltammogram of a bare gold disk working electrode in 0.01 mol L^{-1} PBS (pH 7.4) at a scan rate of 250 mV s^{-1} and a potential step of 1 mV.

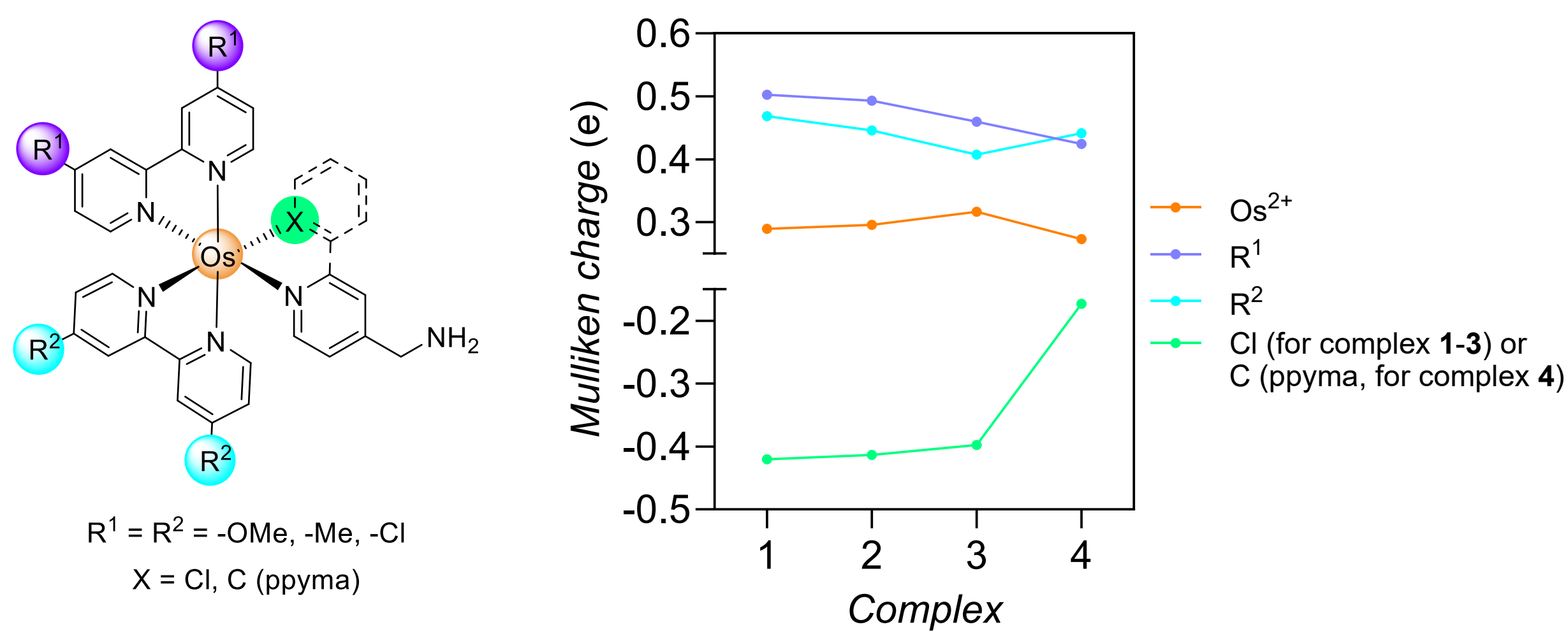

Figure S15. Mulliken charges at the metal center (orange circles), on each substituent of the bpy ancillary ligand (dmb1/dmob1/dclb1: purple circles; dmb2/dmob2/dclb2: blue circles), and on the chloride/carbometalated moiety (Cl/ppyma: green circles) for complexes **1**–**4**.

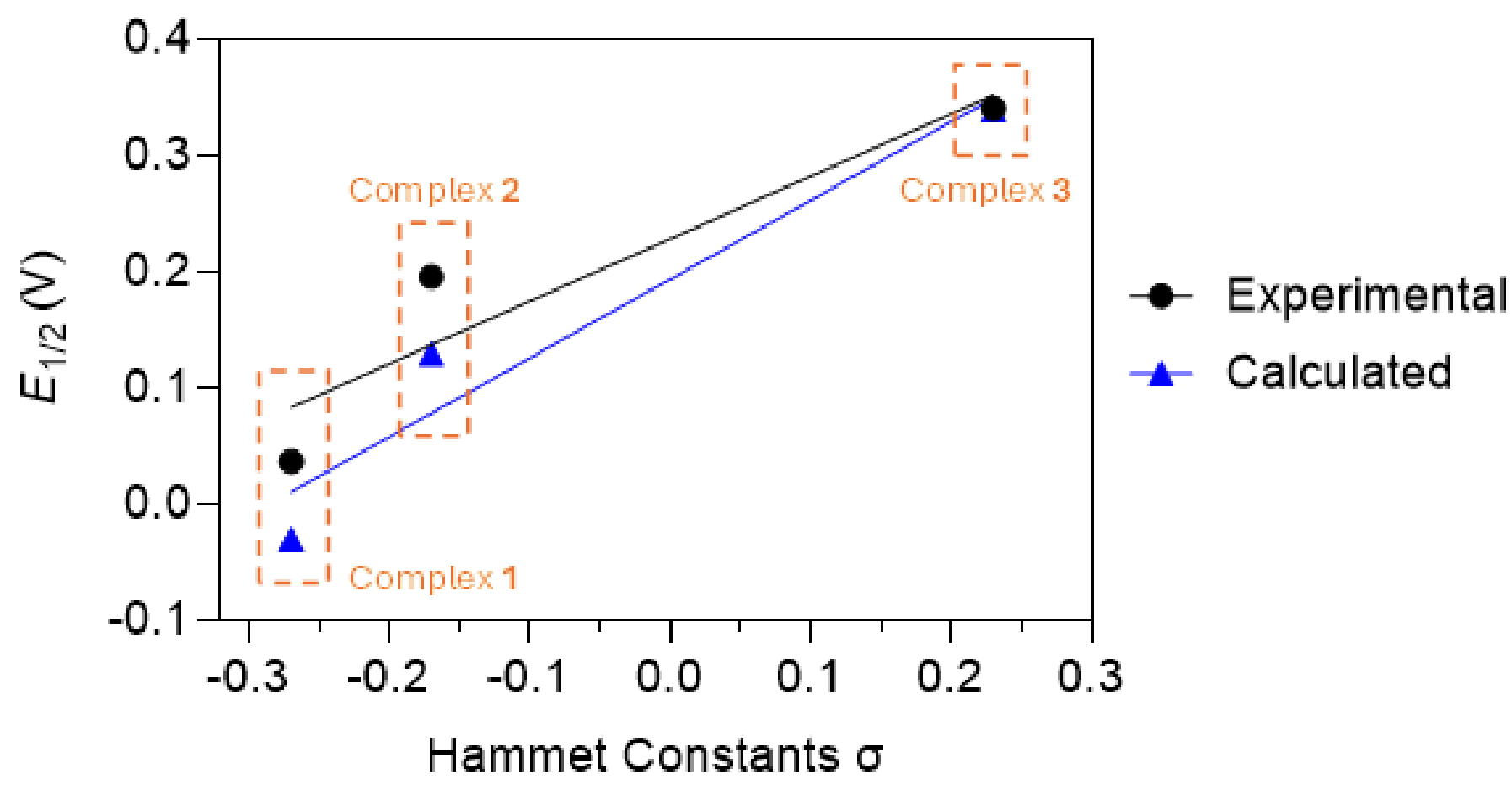

Figure S16. Correlation between the Hammett constant (σ) of the *para*-substituent on the bipyridine *vs* $E_{1/2}$ ($R^2 = 0.88$, slope = +0.20) and $E_{1/2}^{calc}$ ($R^2 = 0.94$, slope = +0.18) of the Os^{2+}/Os^{3+} couples for complexes **1**–**3**.

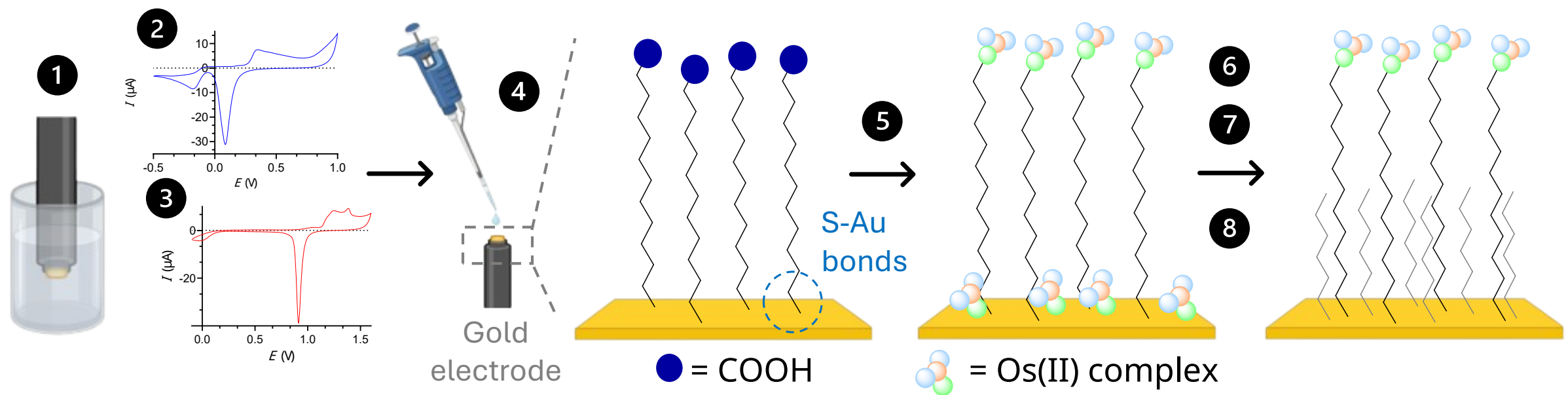

Figure S17. General protocol of the electrodes preparation with self-assembled monolayers and attachment of Os(II) complexes as redox probes by following the next steps: piranha cleaning and polishing (1), electrochemical treatment by CV in 0.1 M NaOH (2) and in 0.5 M H_2SO_4 (3), immobilization of MUA units (4), covalent attachment of Os(II) complex by amide coupling to MUA (5), washing in 1 M H_2SO_4 in acetonitrile (6) and in 6 M guanidinium chloride in 4:1 v/v EtOH/DI water (7), and backfilling with MCH (8).

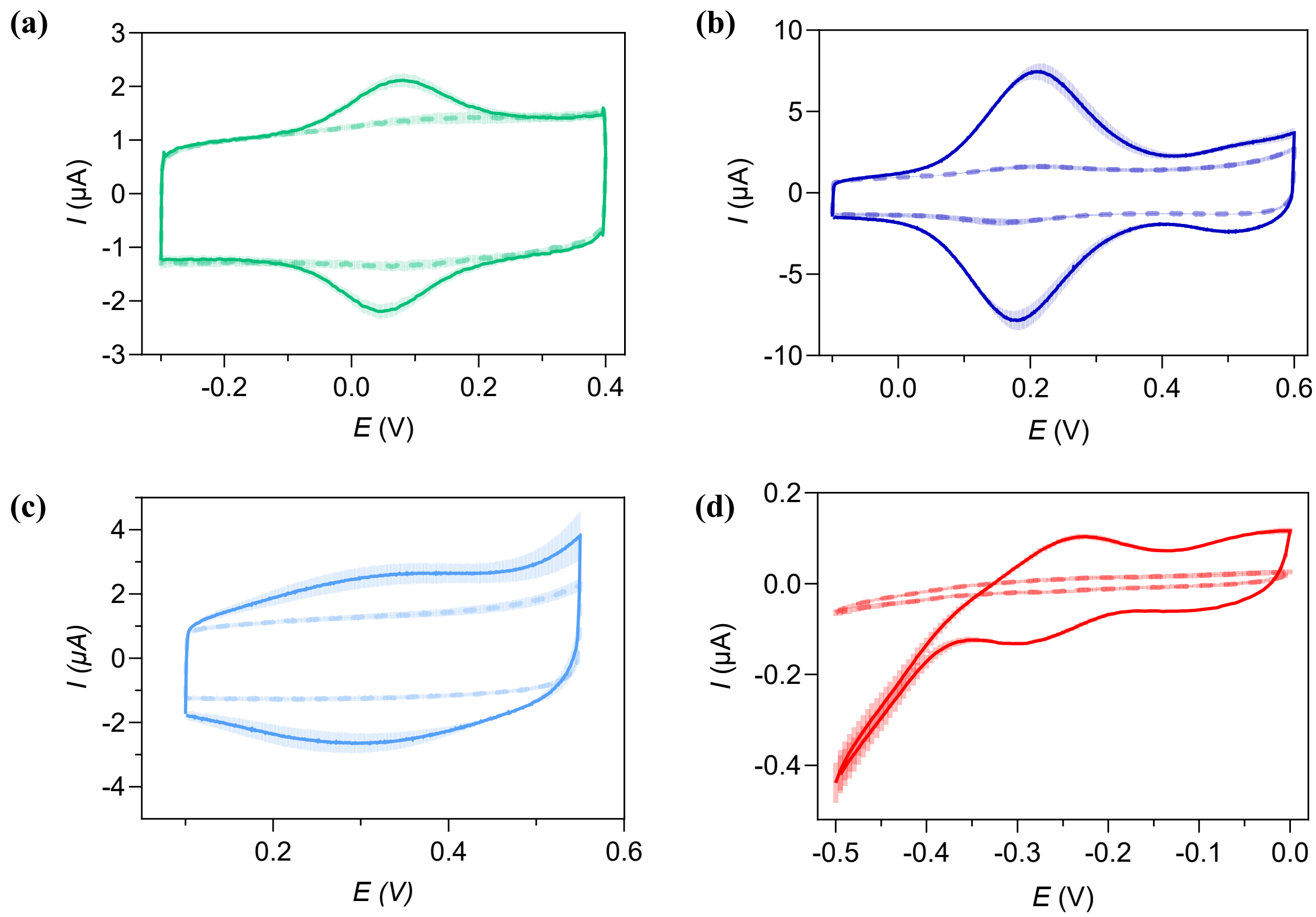

Figure S18. Cyclic voltammograms in PBS (7.4) of (a) Complex **1**, (b) Complex **2**, (c) Complex **3**, and (d) NMB in the presence (solid lines) and absence (dotted lines) of EDC during the coupling reaction to MUA units on gold electrodes (n=3). Measurements were collected using a scan rate of 5 V s^{-1} and a voltage step of 1 mV or, in the case of NMB, a scan rate of 250 mV s^{-1} and a voltage step of 5 mV.

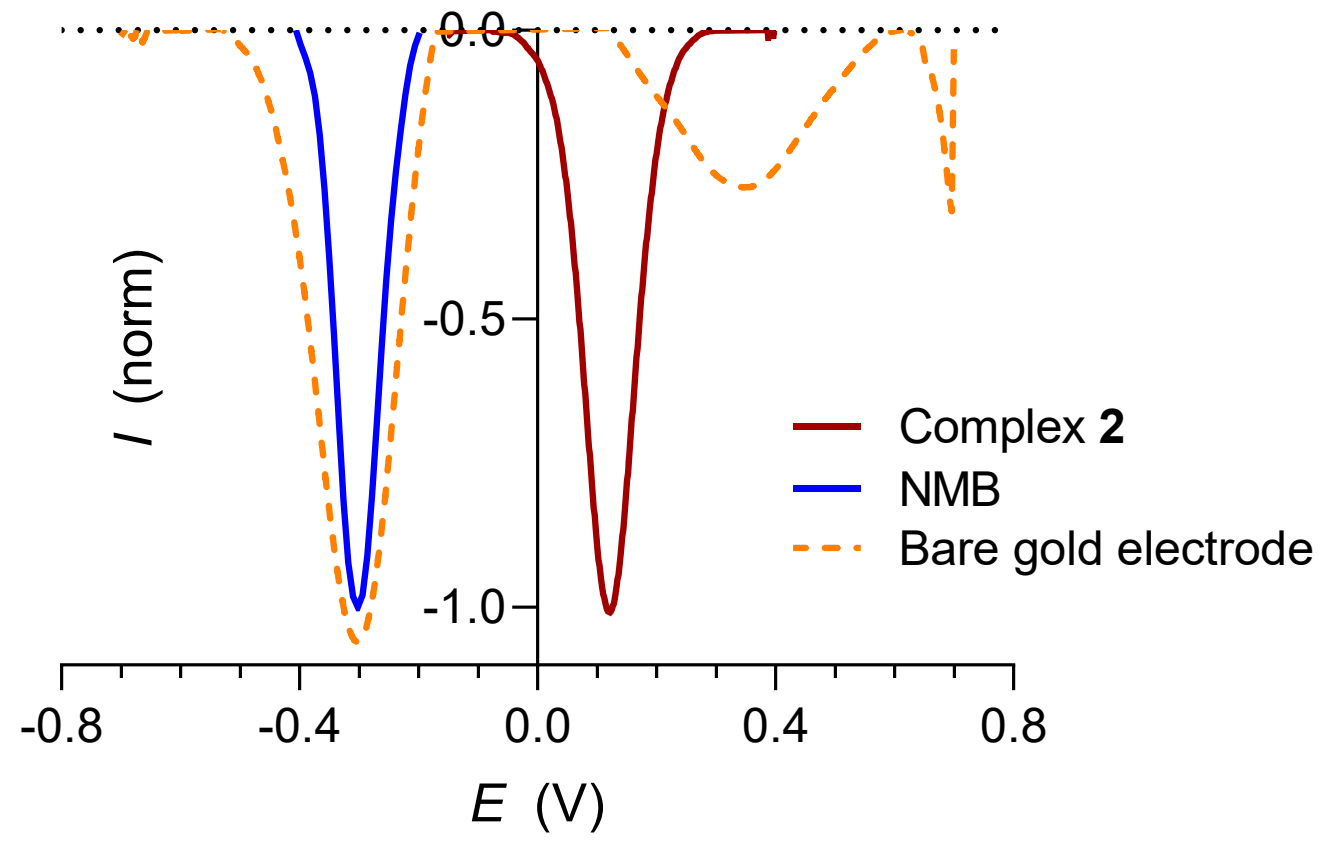

Figure S19. Square wave voltammograms in PBS (7.4) for complex **2**, NMB, and a bare gold electrode. The oxygen reduction process occurs within the potential window of NMB, while gold oxidation is observed between 0.1 and 0.6 V. All measurements were collected using a pulse amplitude of 25 mV, and in the case of complex 2, a potential step of 1 mV, and a frequency of 500 Hz; in the case of NMB, a potential step of 8 mV and a frequency of 25 Hz; and in the case of the bare gold electrode, a potential step of 1 mV and a frequency of 10 Hz.

Table S1. Electrochemical parameters calculated with different ligands in the equatorial position (Cl^-, H_2O, $H_2PO_4^-$) being $E^0_{abs}{}^{calc}$ the absolute calculated potential and $E_{1/2}{}^{calc}$ is the calculated potential against the Ag/AgCl reference electrode (see the Computational methods section in the main text for details concerning their definition).

3

Complex 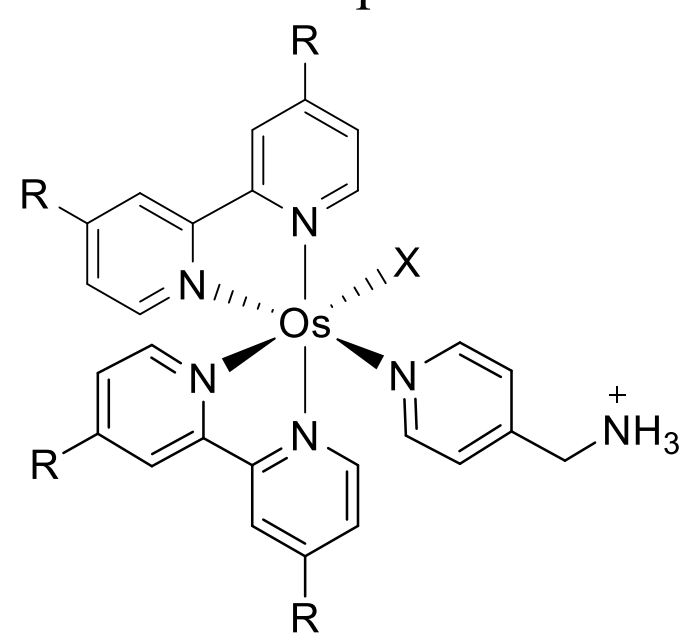		**1** (R = OMe)	**2** (R = Me)	**3** (R = Cl)
$X=Cl^-$	$E^0_{abs}{}^{calc} (V)$	4.606	4.766	4.976
	$E_{1/2}^{calc} (V)$	-0.031	0.129	0.339
	$E_{1/2}^{exp} (V)$	0.036	0.195	0.340
	$E_{1/2}^{calc}-E_{1/2}^{exp} (V)$	-0.067	-0.066	-0.001
$X=H_2O$	$E^0_{abs}{}^{calc} (V)$	5.060	5.299	5.578
	$E_{1/2}^{calc} (V)$	0.423	0.662	0.941
	$E_{1/2}^{exp} (V)$	0.036	0.195	0.340
	$E_{1/2}^{calc}-E_{1/2}^{exp} (V)$	0.387	0.467	0.601
$X=H_2PO_4^-$	$E^0_{abs}{}^{calc} (V)$	4.465	4.561	4.833
	$E_{1/2}^{calc} (V)$	-0.172	-0.076	0.196
	$E_{1/2}^{exp} (V)$	0.036	0.195	0.340
	$E_{1/2}^{calc}-E_{1/2}^{exp} (V)$	-0.208	-0.271	-0.144

Table S2. Absolute SWV peak currents ($|I_p|$), electroactive surface coverage (Γ_{redox}), and coverage-normalized responses of immobilized Os(II) reporters ($|I_p|/ \Gamma_{redox}$) (mean ± SD, n = 3 independent electrodes).

**Reporter**	**$|I_p|$ ± SD / µA**	**Γ_{redox} / pmol cm^{-2}**	**$|I_p|/\Gamma_{redox}$ / µA pmol^{-1} cm^{-2}**
Complex **1**	3.9 ± 0.1	2.5 ± 1.3	1.6 ± 0.9
Complex **2**	71.3 ± 5.7	16.4 ± 8.4	4.3 ± 2.3
Complex **3**	10.8 ± 5.6	4.5 ± 2.0	2.4 ± 1.6
Complex **4**	6.9 ± 3.4	14.4 ± 1.1	0.5 ± 0.2

Determination of thiolated SAM surface coverage by reductive desorption

Desorption of modified gold electrodes was evaluated by reductive desorption in deoxygenated 0.1 mol L^{-1} KOH. Prior to measurement, the electrolyte was degassed by N_2 bubbling for 30 min under stirring in a sealed electrochemical cell. Bare, polished and electrochemically activated gold electrodes were cycled from 0 to −1.3 V at 100 mV s^{-1} for 10 cycles to obtain a stable reference response. Modified electrodes, prepared as described in the Methods section, were then measured under the same conditions for 5 cycles. For modified electrodes, the first cathodic scan was used for reductive desorption analysis. Surface coverage was estimated by integrating the reductive desorption peak and converting the charge to coverage using $\Gamma = Q/nFA$, where Q is the integrated charge, n was taken as 1 electron per Au–S bond, F is Faraday's constant, and A is the electrochemically active surface area of each electrode. When more than one desorption peak was observed, the total charge was obtained by integrating all peaks.

The electrochemically active surface area of each gold electrode was obtained from the activation step in 0.5 mol L^{-1} H_2SO_4 from −0.4 to 1.4 V at 100 mV s^{-1}, by integrating the gold oxide reduction

peak from the last of the 40 cycles to obtain the peak area [A·V], converting it to charge by dividing by the scan rate and using a theoretical charge density of 400 μC cm^{-2} for polycrystalline gold.[3]

Table S3. Thiolated SAM surface coverage determined by reductive desorption (mean ± SD, n = 3 independent electrodes).

**Electrode modification**	**ESA / cm²**	**Q_{des} / μC**	**Γ_{thiol} / nmol cm^{-2}**
MUA	0.07 ± 0.02	21.3 ± 5.1	3.1 ± 0.7
MUA/Complex **1**/MCH	0.11 ± 0.01	17.4 ± 1.2	2.2 ± 0.4
MUA/Complex **2**/MCH	0.074 ± 0.003	16.4 ± 4.2	1.7 ± 0.3
MUA/Complex **3**/MCH	0.077 ± 0.003	17.9 ± 5.7	2.3 ± 0.1
MUA/Complex **4**/MCH	0.083 ± 0.009	14.6 ± 3.3	2.3 ± 0.6

REFERENCES

(1) Shaw, G. B.; Styers-Barnett, D. J.; Gannon, E. Z.; Granger, J. C.; Papanikolas, J. M. Interligand Electron Transfer Dynamics in $[Os(bpy)_3]^{2+}$: Exploring the Excited State Potential Surfaces with Femtosecond Spectroscopy. *J. Phys. Chem. A.* **2004**, *108* (23), 4998–5006. https://doi.org/10.1021/jp0363850.

(2) Han, M.; Yoon, S. H.; Lee, J.; Chung, T. D.; Song, W. J. Redirecting Electron Flows in Glutamate Oxidases by Selective Anchoring of Osmium Complexes. *Chem. Sci.* **2025**, *16* (17), 7433–7441. https://doi.org/10.1039/d5sc00166h.

(3) Trasatti, S.; Petrii, O. A. Real Surface Area Measurements in Electrochemistry. *J. Electroanal. Chem.* **1992**, *327* (1–2), 353–376. https://doi.org/10.1016/0022-0728(92)80162-W.
